# Morpheus: a fragment-based algorithm to predict fold-switching behaviour in proteins across proteomes

**DOI:** 10.1093/bioinformatics/btaf635

**Published:** 2025-11-24

**Authors:** Vijay Subramanian, Rajeswari Appadurai, Harikrishnan Venkatesh, Ashok Sekhar, Anand Srivastava

**Affiliations:** Indian Institute of Science Education and Research, Pune 411008, India; Molecular Biophysics Unit, Indian Institute of Science, Bangalore 560012, India; Molecular Biophysics Unit, Indian Institute of Science, Bangalore 560012, India; Department of Biology, Indian Institute of Science, Education and Research, Tirupati 517619, India; International Business Machine (IBM), Bangalore 560071, India; Molecular Biophysics Unit, Indian Institute of Science, Bangalore 560012, India; Molecular Biophysics Unit, Indian Institute of Science, Bangalore 560012, India

## Abstract

**Motivation:**

Functionally important ‘fold-switching’ proteins, which do not obey the classical folding dogma, are now thought to be widespread. Algorithms that can accurately annotate fold-switching proteins from sequence information can help uncover the true extent of the ‘metamorphome’.

**Results:**

Here, we present Morpheus, a fragment-based classification approach, that works by analysing the diversity of structures within a query protein sequence. Morpheus exhaustively curates and uses fragment structural data from the Protein Data Bank as well as the AlphaFold Protein Structure Database. We employed our algorithm on 57 different proteomes consisting of a total of 601 218 proteins and identified about 10% of these proteins with the ability to fold-switch. Additionally, we provide a web server for Morpheus to test for fold-switching propensities for user-defined sequences (http://mbu.iisc.ac.in/∼anand/morpheus). Besides screening for fold-switching behaviour in proteomes, our work will be useful in de novo design and engineering of such proteins through further experimentation.

**Availability and implementation:**

All codes used for fragment-picking analysis and the proteome-level prediction data for the 57 proteomes are publicly available on Zenodo (https://zenodo.org/records/14837336). The algorithm is also hosted within the web server (http://mbu.iisc.ac.in/∼anand/morpheus).

## 1 Introduction

According to the ‘one-sequence, one-conformation’ paradigm proposed by Anfinsen (1972), the amino acid sequence of a given protein codes for its 3D folded structure required for the protein to perform its intended function. This paradigm has expanded to ‘one-sequence, an ensemble of conformations’ with highly labile but functional intrinsically disordered proteins (IDPs) (Dunker *et al.* 2001). Another branch of proteins that defy this paradigm, although not as conformationally degenerate as IDPs, is metamorphic proteins. Metamorphic proteins are a class of proteins that have the ability to exist in two interconvertible conformations that are stable and significantly different in their secondary or tertiary structures (Murzin 2008, Porter *et al.* 2024). The two conformations correspond to highly dissimilar folds and possibly two different functions that the protein undertakes. Often, there is a biological trigger such as temperature, salt conditions, or the redox state associated with the conformational change that follows (Appadurai *et al.* 2021, Madhurima *et al.* 2021, Ghosh and Jana 2022, Jain and Sekhar 2024).

Some of the well-studied metamorphic proteins include the following: (i) RfaH, a virulence factor found in *Escherichia coli*. RfaH is a multi-domain protein where the inter-domain interaction can modulate the conformation of the C-terminal domain between an alpha-helical hairpin and a beta-barrel conformation. The all-alpha fold of RfaH functions to inhibit its activity, while the all-beta fold enables RfaH to activate transcription and facilitate translation (Burmann *et al.* 2012, Appadurai *et al.* 2021, Artsimovitch and Ramírez-Sarmiento 2022). (ii) KaiB, the cyanobacterial protein switches fold to regulate the phosphorylation/dephosphorylation phases of the circadian clock KaiABC, which allows it to maintain a periodicity of 24 h (Chang *et al.* 2015). KaiB adopts a novel tetrameric quaternary state and only binds to KaiC after fold-switching into a thioredoxin-like fold. Only in its fold-switched state, KaiB binds to KaiC, where the fold-switching contributes to the time delay necessary for the circadian clock. (iii) XCL1, the human lymphotactin, is a metamorphic protein that undergoes a conformational rearrangement from a monomeric α + β chemokine fold to a novel dimeric all-β fold (Tuinstra *et al.* 2008). The canonical chemokine fold performs as an agonist for XCR1 (a G-protein coupled receptor), while the novel dimeric fold binds to surface glycosaminoglycans (Khatua *et al.* 2020). The trigger for the fold-switch in lymphotactin is temperature, and under physiological conditions, both folds are populated. Known metamorphic proteins are believed to be widespread in proteomes of many kingdoms of life and are currently estimated to constitute 0.5–4% of the Protein Data Bank (PDB) (Porter and Looger 2018).

Note the distinction between metamorphic proteins and fold-switching proteins that Porter and co-workers put forth: the main difference between metamorphic and fold-switching proteins is that the secondary structure remodelling events of fold-switchers can be either reversible or irreversible, but that of metamorphic proteins must always be reversible (Kim and Porter 2021). Hence, metamorphic proteins form a subset of fold-switching proteins. All the metamorphic proteins in the proteome can be called the ‘metamorphome’. Estimates have been made, but it is unclear how much of this class of proteins is present in the proteome overall. Protein sequences must be computationally screened for possible metamorphic behaviour and then validated using experimental methods in order to uncover the metamorphome in its entirety. Previous attempts have been made to predict metamorphic behaviour in proteins using protein sequence information. One of the approaches, from Chen and co-workers, is guided by the hypothesis that metamorphic protein sequences would lead to degenerate secondary structure predictions (Chen *et al.* 2020). Leveraging this degeneracy or confusion in predicting the secondary structure from the sequence data, the authors devised the diversity index, a metric to quantify the confusion of assigned secondary structures by structure prediction tools. Attempts have also been made to predict those fold-switching proteins that undergo alpha-helix to beta-sheet transition using sequence information alone to a high degree of accuracy (Mishra *et al.* 2021). This particular type of fold-switching protein, where there is a major switch from alpha-helical conformation to a beta-sheet, constitutes a relatively small portion of the known metamorphic proteins overall. Algorithms that tackled a parallel problem of predicting sequence-similar fold-switchers has also been developed (Kim *et al.* 2021). Here, two different proteins with high levels of aligned similarity were assessed to see if they assumed the same fold or different ones. In contrast to fold-switching proteins, the sequence-similar fold-switching proteins described above remodel their secondary structures in response to mutations. Precise successive point mutations in protein sequences can lead to entirely different protein folds (He *et al.* 2012).

Another set of approaches that are designed to address a broader problem are methods that employ AlphaFold suite of software to study conformational changes (Jumper *et al.* 2021, Osman 2023, Schafer and Porter 2023, Bryant and Noé 2024, Wayment-Steele *et al.* 2024). It is unclear whether the results obtained from AlphaFold-based techniques are generative or based on memory; there is evidence supporting both sides of the argument (Chakravarty *et al.* 2023  2024, Tyree and Kim 2024). When these methods are used in conjunction with high-throughput bioinformatic approaches, good results can be achieved compared to the results obtained individually. While it is worthwhile trying to predict fold-switching proteins using sequence information alone, the approach falls short at making proteome-level predictions due to a high false negativity rate. Methods involving biasing AlphaFold to predict the alternate conformations of metamorphic proteins are often unreliable, resource-intensive and of high time complexity. Here, we present Morpheus, a fast fragment-based predictor of fold-switching proteins that can achieve an average cross-validation accuracy of 84.8% with Matthew’s correlation coefficient of 0.698. To achieve this, we have implemented a Trie-based search to tremendously expedite the fragment-picking performance for large proteomes. We employ our algorithm on 57 different proteomes and present the results here. Finally, we provide a web server for using Morpheus to predict fold-switching protein sequences that were not covered in the dataset presented in this paper (web server link: Morpheus). Additionally, from our database, we shortlist a few candidate fold-switching proteins that are involved in crucial biological processes and are highly likely to switch folds. These shortlisted proteins can be excellent candidates for further experimental work.

## 2 Materials and methods

### 2.1 Training dataset

The set of known fold-switching proteins and proteins that are highly likely to have a single fold is used as a training dataset. This data set has been borrowed from existing curated literature (Porter and Looger 2018, Chen *et al.* 2020). Additionally, the training dataset is expanded to include the designed metamorphic proteins as well (Solomon *et al.* 2023). In total, 189 fold-switching sequences and 198 monomorphic protein sequences are considered for the training dataset (see Tables 1 and 2, available as supplementary data at *Bioinformatics* online). Although we choose to work with a training dataset of known fold-switching proteins, our classifier can also be used to select candidate metamorphic proteins for a couple of reasons: (a) There are only a handful of truly metamorphic proteins, and it is unlikely to train a model on such limited data meaningfully. (b) Our model predicts those metamorphic proteins where there is a large change in the secondary structure of the two conformations with high confidence. Hence, when predictions are made, analysing the highly confident hits could help in searching for potential metamorphic proteins as well.

**Table 1.** The diversity metrics of known metamorphic proteins and proteins that are highly likely to be monomorphic are ordered according to decreasing diversity scores.

Protein	Diversity	Entropy	Substitution	Uncertainty
Metamorphic				
RfaH	2.08	0.78	0.09	0.26
XCL1	1.78	0.56	0.02	0.46
KaiB	1.73	0.63	0.25	0.09
MAD2	1.66	0.55	0.17	0.05
Designed protein	1.58	0.44	0.16	0.32
HIV-RT1	1.53	0.43	0.13	0.02
IscU	1.47	0.36	0.13	0.74
MinE	1.47	0.43	0.09	0.20
Selecase	1.45	0.40	0.15	0.26
CLIC1	1.37	0.27	0.18	0.04
Monomorphic				
Luffaculin	1.53	0.42	0.08	0.41
Histone H3	1.50	0.51	0.05	0.00
Beta-lactamase	1.44	0.35	0.17	0.01
GFP	1.39	0.34	0.15	0.02
Chymotrypsinogen	1.38	0.33	0.21	0.14
Ribonuclease	1.29	0.22	0.09	0.55
Trypsin	1.27	0.26	0.08	0.01
Ubiquitin	1.26	0.28	0.09	0.00
Caspase-3	1.10	0.11	0.05	0.01

**Table 2.** The validation accuracy measures for Morpheus and the method by Liwang’s group averaged across the cross-validation partitions are mentioned.^a^

Measure	Morpheus	Chen *et al.*
Sensitivity	0.79 (0.08)	0.63 (0.09)
Specificity	0.90 (0.06)	0.78 (0.06)
Precision	0.89 (0.08)	0.81
Accuracy	84.8% (6.1)	69.8% (1.5)
*F*1 score	0.83 (0.08)	0.71
MCC	0.698 (0.12)	0.355 (0.104)

a The numbers in the parentheses are the standard deviation for these values calculated across the cross-validation partitions. The values for Chen *et al.* directly taken from the values mentioned in the publication; precision and *F*1 score were inferred from the other values, hence we could not provide a standard deviation value.

### 2.2 Fragment picking

**Figure 1.**
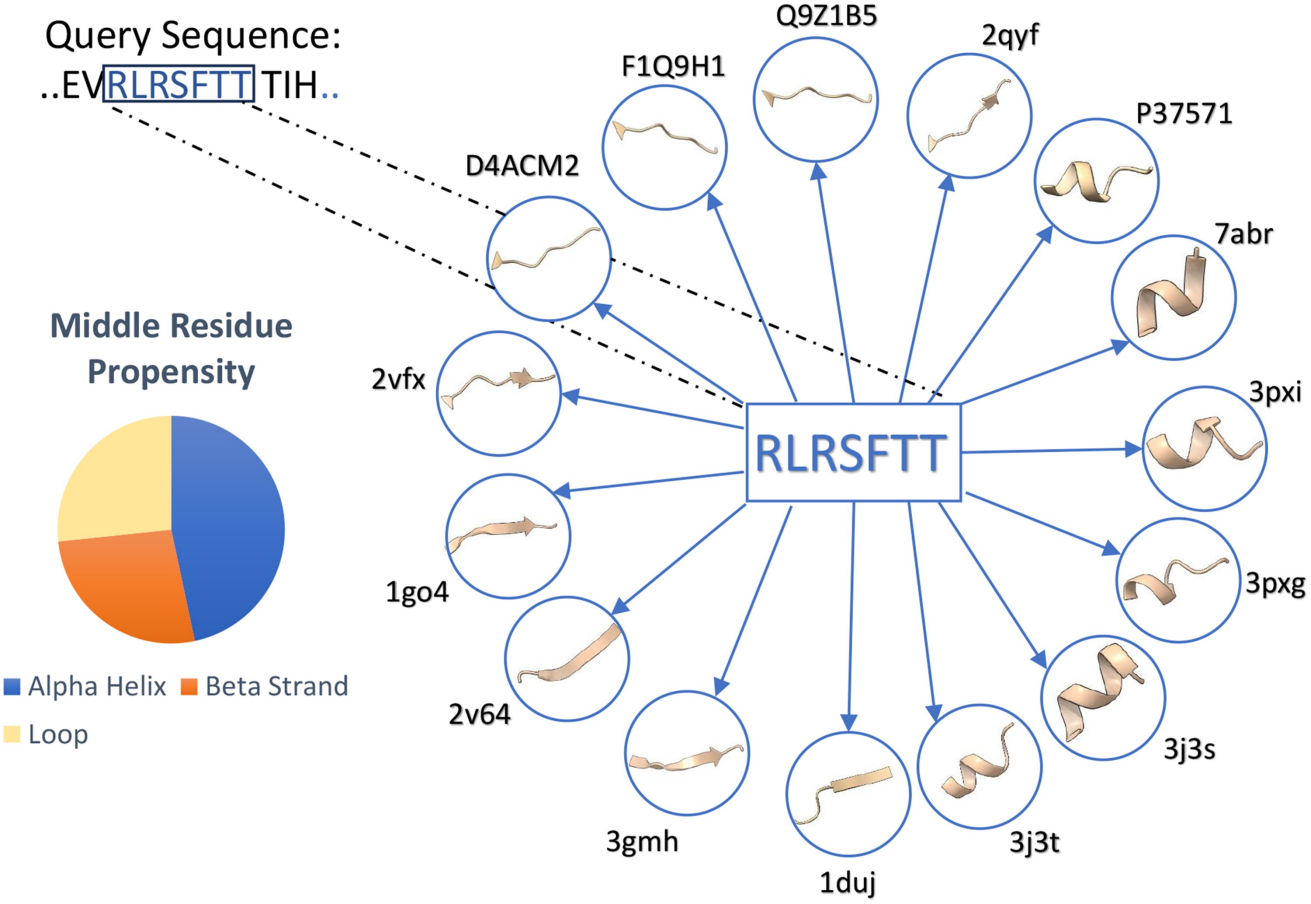
Fragment-picking schematic. The query sequence fragment shown is a part of the metamorphic protein MAD2 and the cartoon representation of the 3D structures of proteins containing the fragments is shown in the circles. The PDB ID and the AlphaFold Accession number are indicated in the representative hits. Figure 1, available as supplementary data at *Bioinformatics* online, shows similar schematics for other fragment sequences.

The long query sequence is first broken into fragments using a sliding window of sequence size 7. Each fragment from the query sequence is searched for identical fragments across a database of protein sequences containing around 200 000 redundant proteins from PDB (Berman *et al.* 2000) and around 1 million protein structure predictions from AlphaFold2. Given our fragment-picking database size, 7-mer fragments give the optimal number of hits while maintaining the secondary structure diversity of the hits. Once the hits are obtained, the secondary structure of the hits, which is assigned from the original structure using DSSP (Kabsch and Sander 1983), is extracted for the purpose of scoring. The hits are filtered to remove those fragments whose middle residue is an unobserved residue or has a local pLDDT score of <70 in the case of experimentally solved structures and computationally solved structures, respectively. As we perform the fragment search on a redundant PDB database, many of the hits occurring from different protein structures may all be pertaining to the same protein sequence. Hence, to eliminate the over-representation of proteins that have multiple redundant structures with the same sequence deposited in the PDB, we use a sequence clustering algorithm to cluster the database with a sequence identity threshold of 100% using the tool MMseqs2 (Steinegger and Söding 2017) (see Algorithm 1, available as supplementary data at *Bioinformatics* online). Figure 1 shows the fragment-picking schematic for one 7-mer fragment.

### 2.3 Scoring metrics

**Figure 2.**
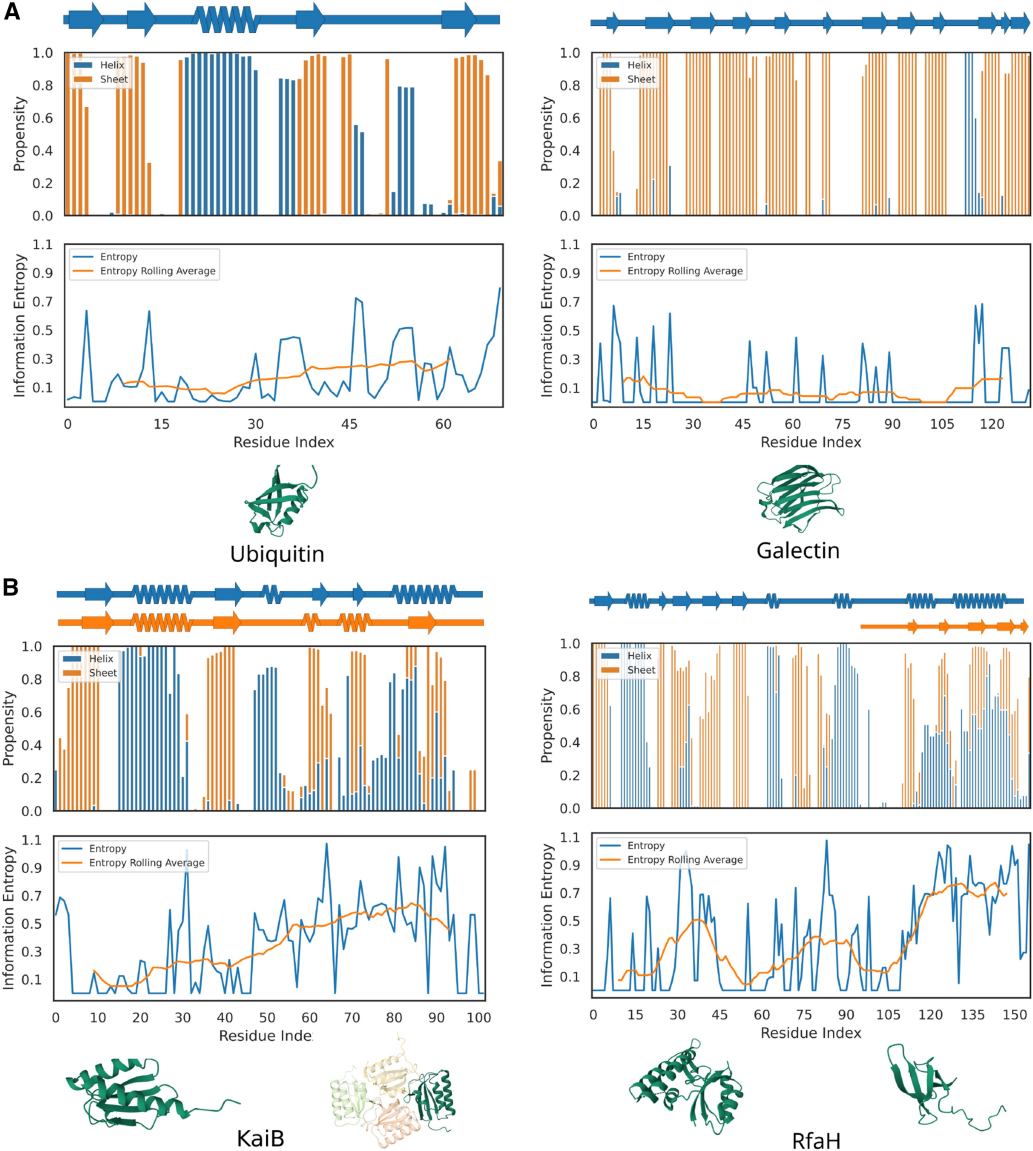
Diversity metrics capture regions of fold-switching. The secondary structure diagram annotated through an experimentally solved structure (top), secondary structure propensity plot (middle) calculated via fragment picking by Morpheus and entropy scores with respect to residue number (bottom) are plotted for (A) Monomorphic proteins Ubiquitin and Galectin (PDB ID: 1UBQ and 2NN8), (B) Known metamorphic proteins Kaib and RfaH (PDB ID: 5JYT and 2QKE, 2OUG and 2LCL). The secondary structure diagrams were made using SSDraw (Chen and Porter 2023).

A particular fragment is scored based on the different secondary structures of the hits obtained from the database. We hypothesized that local interactions within the fragment of seven residues play a major role in determining the secondary structure of the middle residue. Guided by our hypothesis, we opted to analyse the secondary structure of the middle residue of the picked fragments. Fragment-based *ab initio* structure prediction tools like Rosetta (Gront *et al.* 2011) also choose to work with the secondary structure of the middle residue from picked fragments. Therefore, the secondary structure of the middle residue is retrieved from each fragment hit obtained from our database. Since fold-switching proteins adopt two distinct conformations, often having a change in secondary structure, the metric we choose should be able to quantify the ability of a fragment sequence to adopt distinct secondary structures. Multiple consecutive fragments displaying this ability would, in turn, aid the whole protein sequence to switch folds. Following this thought, we consolidated four different metrics: (i) diversity index, (ii) substitution score, (iii) information entropy, and (iv) uncertainty score. The diversity index (Chen *et al.* 2020) and substitution matrix-based scoring methods (Porter and Looger 2018) have already been used for the classification of metamorphic proteins. Information entropy and uncertainty score are metrics that we have introduced for the purpose of classification of metamorphic proteins. Diversity index and information entropy are defined as follows:


$$\begin{array}{cc} & \text{DI}={\left(h^{2}+e^{2}+l^{2}\right)}^{-1}\\ \;\text{Entropy} & =-h\;\text{log}(h)-e\;\text{log}(e)-\mathrm{llog}(l) \end{array}$$

where *h*, *e*, and *l* are the calculated propensities of the middle residue to be in helix, sheet, and loop, respectively. The secondary structure propensities for each 7-mer are calculated from the hits obtained from the database by assessing what fraction of those hits have middle residues existing in helix, sheet, and loop secondary structure. Refer to Section 1.6.1, available as supplementary data at *Bioinformatics* online, for more details. Figure 2 displays the diversity metrics for a few metamorphic and monomorphic proteins. Figure 2 shows that the region where the entropy score remains consistently high correlates well with the region of the protein that actually switches fold in the case of metamorphic proteins: KaiB and RfaH and remain low overall for the monomorphic proteins shown. Figure 2, available as supplementary data at *Bioinformatics* online, portray the diversity metrics for other known metamorphic proteins such as RfaH, KaiB, IscU, MAD2, Lymphotactin, Selecase, MinE, CLIC1, HIV-RT1. We also show data for a designed protein Sa1V90T (PDB ID: 8E6Y) from John Orban and co-workers (Solomon *et al.* 2023).

We implemented a substitution matrix-based scoring method in order to evaluate the ability of the fragments to adopt different secondary structure conformation. The fragment hits are pairwise aligned and scored using the following substitution matrix. Refer Section 1.6.2, available as supplementary data at *Bioinformatics* online, for more details


$$\begin{array}{c} \frac{1}{7}\left[\begin{array}{cccc} \text{ref}/\text{replace} & H/I/G & E/B & T\\ H/I/G & 0 & 0.7 & 0.3\\ E/B & 0.7 & 0 & 1\\ T & 0.3 & 1 & 0 \end{array}\right] \end{array}$$

We also sought to quantify the confidence in the calculated diversity metrics for each sliding window based on the number of hits per fragment. Using Hoeffding’s inequality (Section 1.6.4, available as supplementary data at *Bioinformatics* online), we modelled uncertainty as an exponential function of sample size


Uncertainty=exp (−0.5×#of hits from unique clusters)

We hypothesized that contiguous regions with high entropy suggest a higher likelihood of fold-switching. To capture these regions, we applied a rolling average to the scores, with the window width optimized using training data (Table 3, available as supplementary data at *Bioinformatics* online). A step function, shown below, defines the rolling window


$$\text{Window}\;\text{width}\;(\text{WW})=\{\begin{array}{c} 8\;\text{length}(\text{protein}\;\text{sequence})\leq 25\\ 18\;\text{length}(\text{protein}\;\text{sequence})>25 \end{array}$$

**Table 3.** List of potential candidates for fold-switching proteins.

Uniprot ID	Organism	Protein Name	Length	Confidence	Uncertainty
Q9Y2Y1	*Homo sapiens*	DNA-directed RNA polymerase III subunit RPC10	108	4.83	0.18
Q4CMH7*	*Trypanosoma cruzi*	Guanine nucleotide-binding protein subunit beta-like protein	102	4.80	0.44
P26995	*Pseudomonas aeruginosa*	Transcriptional anti-antiactivator ExsC	145	2.82	0.43
Q9UI95	*Homo sapiens*	Mitotic spindle assembly checkpoint protein MAD2B	211	2.47	0.01
A0A1D6P714*	*Zea mays*	Extensin-like protein	129	2.27	0.61
A0A804PFY2*	*Zea mays*	Photolyase/cryptochrome alpha/beta domain-containing protein	162	1.86	0.45
A0A3P7DY23*	*Wuchereria bancrofti*	S1 motif domain-containing protein	258	1.23	0.39
Q4DL46*	*Trypanosoma cruzi*	Copper-transporting ATPase-like protein, putative	263	1.03	0.25
B8A185*	*Zea mays*	Secreted protein	112	0.72	0.34

* do not have any experimentally solved structures deposited in the PDB as of when this article was written.

The final score that is used as the input vector for the classification model is all the aforementioned scores together. The four scores together are used as the feature space for the classification model, as shown below:


$$\begin{array}{c} \text{Final}\;\text{Score}=\left(\begin{array}{c} \max\left(\frac{1}{WW}\sum_{j=0}^{j<WW}{\text{Diversity}}_{i+j}\right)\\ \max\left(\frac{1}{WW}\sum_{j=0}^{j<WW}{\text{Entropy}}_{i+j}\right)\\ \max\left(\frac{1}{WW}\sum_{j=0}^{j<WW}{\text{Substitution}}_{i+j}\right)\\ \mathrm{mean}\left(\text{uncertainty}\right) \end{array}\right) \end{array}$$

We found that using residues other than the central one in the 7-mer yielded similar accuracy and were within one standard deviation of each other, with the third residue performing best (Table 4, available as supplementary data at *Bioinformatics* online). Moving forward, we used the third residue for classification. In the final scoring, index *i* ranges from 3 to L − 7 + 3, where *L* is the sequence length. If no hits are found for a fragment, we assign default scores: diversity = 1, entropy = 0, substitution = 0, and uncertainty = 1.0, to minimize false positives when we have no data to estimate the diversity of the fragment. See Section 1, available as supplementary data at *Bioinformatics* online: details of materials and methods for an elaborate explanation of the scoring metrics.

### 2.4 Prediction model

**Figure 3.**
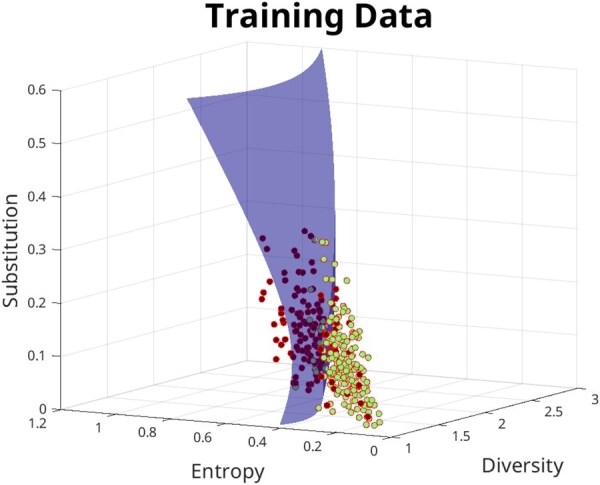
The training dataset with 3/4 features. The training dataset of known fold-switching proteins and highly likely monomorphic proteins are plotted in the feature space of the variables diversity score, entropy score, and substitution score. The points in Red depict fold-switching proteins and the points in Green depict monomorphic proteins. The decision boundary shows the optimized SVM model output when a quadratic kernel is used.

A final prediction model is constructed using the aforementioned features. The training data is randomly split according to a six-fold cross-validation scheme. Upon performing a six-fold cross-validation, an SVM model with a polynomial kernel of degree 2 is found to be the model that has the highest average validation accuracy while also maintaining a lower model complexity. Figure 3 shows the decision boundary that is finally obtained after optimizing the support vector machine using a Bayesian optimization scheme for the SVM hyper-parameters. Table 1 shows the diversity metrics for the known and experimentally verified metamorphic proteins and a few monomorphic proteins.

Figure 3a–f, available as supplementary data at *Bioinformatics* online, show how the decision boundary varies as the model is re-trained across each of the six partitions and corresponding confusion matrices. Figure 3g, available as supplementary data at *Bioinformatics* online, shows the ROC (receiver-operating characteristic) curve for the final optimized SVM model. The four features are plotted in a parallel coordinates plot to see the mutual variation in all four coordinates with reference to each other (see Fig. 3h, available as supplementary data at *Bioinformatics* online). The final prediction model uses all four of the features to make new predictions.

### 2.5 Work flow of the algorithm

**Figure 4.**
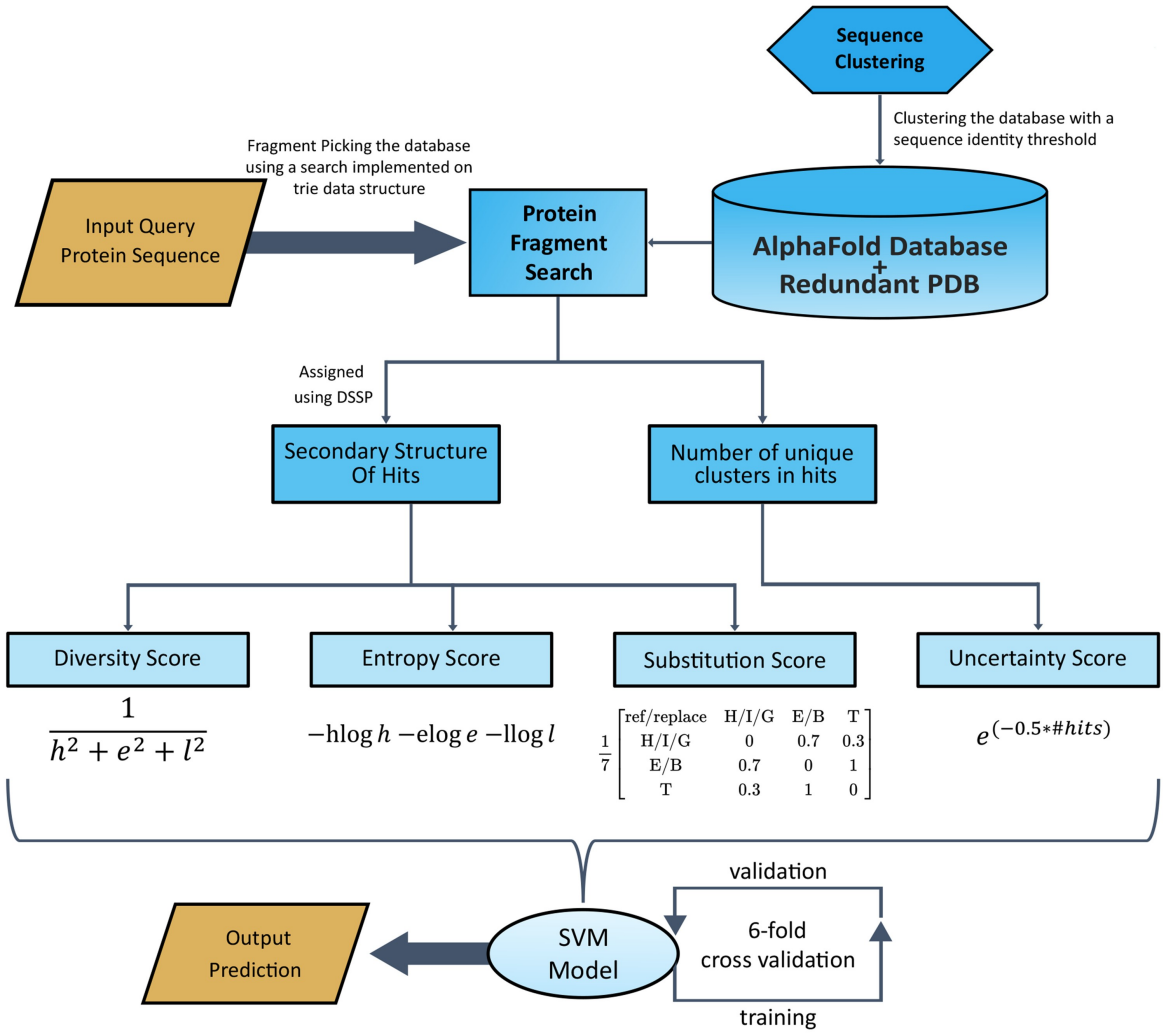
Workflow diagram. Workflow is depicted starting with the fragment sequence search across the database. The hits are then scored for the diversity of secondary structures shown across different structures through four different scoring techniques. An SVM model with a quadratic kernel is employed with the scores as the features. The SVM model is trained and a six-fold cross-validation scheme is applied. Finally, a binary output prediction is made using the trained SVM model.

The workflow of the algorithm is depicted schematically in Fig. 4. The flow of information starts from the input query sequence, which is then used to perform fragment sequence search on the database, and then scored and fed into the SVM model. A binary output is produced, indicating whether the prediction for the query protein is fold-switching or monomorphic.

## 3 Results and discussion

### 3.1 Fragment structure-based predictions outperform purely sequence-based algorithm

Having constructed a classifier to predict the fold-switching behaviour in proteins using protein sequence information alone, we applied our algorithm to predict fold-switching proteins across 57 proteomes from different organisms. We also choose a few candidate fold-switching proteins from our predictions that are involved in crucial biological processes and are highly likely to switch folds based on a few criteria that are mentioned further in the results subsection.

Our Model can predict fold-switching proteins with an average cross-validation accuracy of 84.8% and Matthew’s correlation coefficient of 0.698. Our method outperforms existing sequence-based metamorphic protein predictors while also maintaining a low false-positivity rate of 9.6%. Table 2 depicts the different measures of accuracy for the quadratic SVM model and existing sequence-based predictor of metamorphic proteins. Such measures of accuracy open up the exciting possibility of predicting fold-switching proteins at the proteome level.

Furthermore, plotting the diversity metrics and the secondary structure propensities at each residue allows us to gauge the approximate region that undergoes the fold-switch. For example, in Fig. 2, residues around 51–100 of the known metamorphic protein KaiB show a contiguous region with a high entropy score. These residues overlap well with the experimentally shown region of fold-switching. This level of residue-wise information is expected to be useful when experiments are performed on candidate fold-switching proteins. Additionally, the nature of the fold-switch that may happen in the putative hits can also be inferred by analysing the secondary structure propensity plot of the candidate protein sequence.

### 3.2 Predictions across proteomes reveal varying widespread fold-switching in proteins

As we now have an algorithm that is able to predict the fold-switching behaviour in proteins with high accuracy, we sought to make predictions on the proteomes of many different organisms. We have run our model on 57 different proteomes chosen from all kingdoms of life, from unicellular prokaryotes to multicellular eukaryotes. Analysing different proteomes would have distinct advantages; e.g. analysing an archaean species would be beneficial, considering that we would be working with a proteome of smaller size, and proteins that are functionally similar to eukaryotic proteins, making them excellent models for understanding complex eukaryotic machinery without the added complexity of a eukaryotic cell. We further noticed that viral proteins were more frequently populated in the training dataset of known fold-switching proteins, so we decided to make predictions with our algorithm on a consolidated database of viral proteins. All the data for the 57 proteomes obtained from our predictions are made publicly available on the following Zenodo repository (https://zenodo.org/records/14837336). Furthermore, we offer a web server to execute the algorithm for proteins that are not included in our local runs (Morpheus).

**Figure 5.**
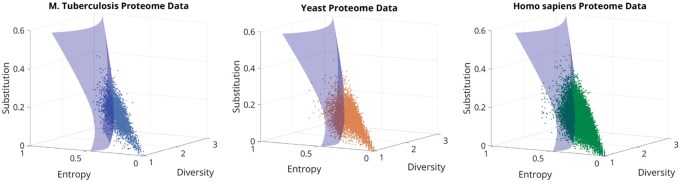
Distribution of proteins from three proteomes on the feature space overlayed with the decision boundary enabling new predictions of fold-switching proteins. 3995, 6060, and 20634 proteins from tuberculosis, yeast, and the human proteome, respectively, are plotted as a scatter plot in the feature space of variables diversity score, entropy score, and substitution score. The SVM decision boundary is also plotted along with the data points. 831 (20.8%), 1289 (21.27%), and 4949 (23.98%) proteins from tuberculosis, yeast, and the human proteome, respectively, are predicted to switch folds by our algorithm.

Figure 5 shows the diversity metrics for all the proteins from the *Mycobacterium tuberculosis*, yeast, and human proteomes. Figure 4, available as supplementary data at *Bioinformatics* online, also display the proteome-level data for a couple of other proteomes. The full dataset for the 57 proteomes is provided in the Zenodo server as an Excel sheet for each proteome. Once we plot the diversity metrics for all the proteins, overlaying the decision boundary helps us make binary predictions of the fold-switching behaviour of those proteins, along with a confidence score based on how far away from the decision boundary the points lie. We also notice that of the proteins that are predicted fold-switching by our algorithm, a good number of them are IDPs. This is expected as our algorithm quantifies the diversity in secondary structures adopted by a particular fragment in the query protein with the help of a structural database. It so happens that IDPs that occupy a highly varying secondary structure across the different deposited structures are reported as containing a high diversity of secondary structures. When analysing the final prediction set, each protein is filtered on the basis of two conditions: (i) If the region predicted to be fold-switching has more than 50% overlap with the consensus of regions predicted to be disordered by the two disorder predictors IUPRED2A (Mészáros *et al.* 2018) and Metapredict (Lotthammer *et al.* 2024), then they are not considered in the final set. (ii) The proteins are further filtered using the protein domain information on UniProt web server. If the fold-switching region has no overlap with protein domain annotation, then they are not considered in the final set (See Section 1.5, available as supplementary data at *Bioinformatics* online, for more details).

**Figure 6.**
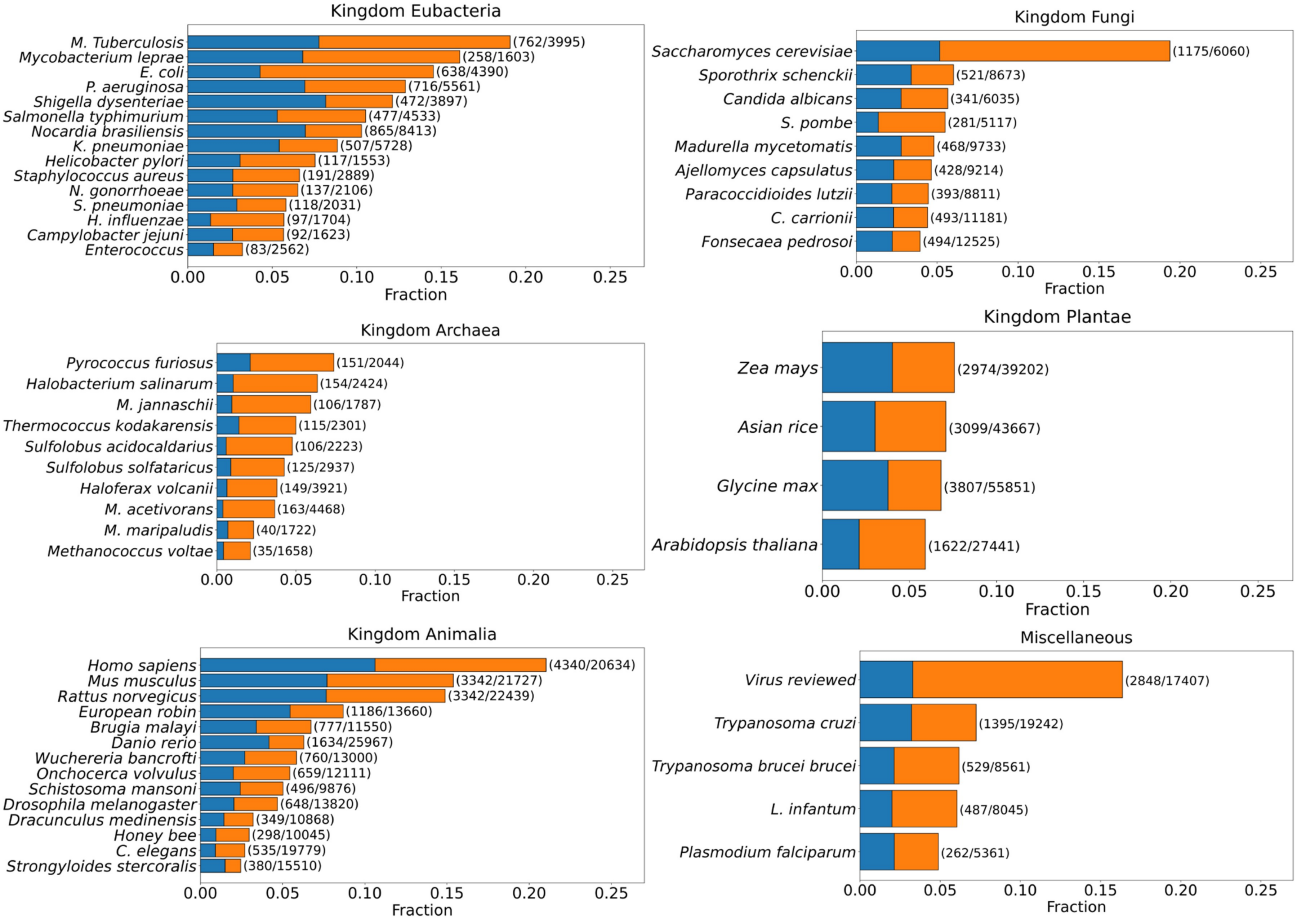
The distribution of fold-switching proteins predicted by Morpheus across different proteomes with and without domain annotation. The distribution of fold-switching proteins predicted by Morpheus after filtering using the criteria mentioned, displayed in the stacked bar graph. The bars that are Blue correspond to the proteins whose fold-switching region overlaps with an annotated domain from UniProt, while the one in Orange represents the proteins which do not have any domain annotation on UniProt. For each bar, numbers in parentheses indicate predicted fold-switching proteins / total proteins in the proteome used for the prediction. The plots are segregated into subplots based on the different kingdoms of life, and the final subplot titled miscellaneous contains viral species (consolidated database of manually reviewed proteins from viral proteomes) and from other kingdoms such as Excavata and Protista.

Figure 6 shows what fraction of the proteomes are predicted to switch folds by our algorithm. These numbers represent the predictions that have cleared the filtering process. The bar plot shown is a stacked bar plot, with blue bars representing those proteins whose fold-switching region falls on an annotated protein domain and orange bars representing proteins that do not have any annotated protein domains. The set of proteins that are predicted to be fold-switching with high confidence is a promising source for recruiting potential candidates that can be further tested for fold-switching behaviour through experimentation. In the following section, we provide a set of proteins that are picked from the prediction set, that show a strong helical and sheet propensity according to Morpheus’ fragment-picking algorithm.

### 3.3 Shortlisted candidate proteins for possible further investigation

In the search for strong candidate fold-switching proteins, we selected a few proteins from the filtered prediction set with the following criteria in mind: (i) The protein should have an annotated protein domain and the fold-switching region should overlap with this domain. (ii) The protein should have a range of residues where the helix and sheet propensity values are both non-zero for a contiguous region indicating the potential ability to fold-switch. Based on these criteria, we select a few of these proteins and present them here in this section.

Table 3 lists a few proteins that are predicted to be fold-switching and are good candidates to be considered for further experimentation. The list contains a protein sequence corresponding to MAD2B, which is related to already known metamorphic protein MAD2A. The protein sequence of MAD2B is not similar to its metamorphic counterpart. The metamorphic behaviour of this protein has not yet been studied.

So far, only one protein that is part of the circadian rhythm has been experimentally shown to be metamorphic in nature (Chang *et al.* 2015). Since circadian rhythm is periodic and helps the organism in synchronizing with the time of the day, it would be quite an exciting find, if a new metamorphic protein joins the circadian rhythm. In our dataset, one of the promising protein predictions involved in the circadian rhythm is ‘Photolyase/cryptochrome alpha/beta domain-containing protein’ which is part of the plant proteome of Maize. In plants, cryptochrome acts as a Blue light receptor to entrain circadian rhythms. They also mediate a variety of light responses, such as the regulation of flowering and seedling growth (Mei and Dvornyk 2015). However, there are no solved structures for this particular protein in maize. Hence, this is a possible avenue for further research. See Fig. 5a–i, available as supplementary data at *Bioinformatics* online, where the diversity metrics are plotted for the selected few predictions listed in the article. The plots contain calculated helix and sheet propensities, the entropy scores, the diversity scores, and substitution scores at each sliding window. The uncertainty score at each fragment is also taken into a rolling average and overlayed on the diversity score.

### 3.4 Scope and limitations

Conventionally, the two conformations of fold-switching proteins are experimentally validated by protein structure determination techniques like NMR and cryo-electron microscopy, or large changes in the secondary structure of proteins can be found through circular dichroism (Porter *et al.* 2022). These methods can prove to be resource-intensive and time-consuming. To add to that, the trigger for fold-switching may not be known beforehand, making the detection of fold-switching proteins further difficult. Therefore, providing a way to systematically filter out monomorphic proteins and to find candidate fold-switching proteins only using the sequence information of the protein is highly useful. Our work is the first implementation of a proteome-wide fold-switching protein predictor using protein sequence information. Morpheus allows for the prediction of proteins whose structure may not be deposited in the PDB. Hence, our method is a step in the direction of expanding the known set of fold-switching proteins. Besides fulfilling a researcher’s curiosity, analysing the human proteome for fold-switching proteins can stand a chance at helping global healthcare and improving therapeutics by furthering the understanding of protein fold-switching and the different physiological triggers involved within. Fold-switching proteins associated with a number of diseases have already been identified (Lei and Takahama 2012, Jain *et al.* 2014, Li *et al.* 2018, Costello *et al.* 2022). Broadening the reservoir of predicted metamorphic proteins help in paving ways for new research.

Although we are able to make high-throughput predictions of fold-switching behaviour in proteins to a good accuracy, our algorithm falls short at making sensitive predictions when the sequences differ by point mutations. For example, E48A in RfaH has been shown to disrupt the inter-domain contact and thus favour all-beta conformation (Burmann *et al.* 2012, Dishman and Volkman 2018). So, while analysing highly similar homologous sequences, the results obtained from our algorithm may be questionable. Further, since our algorithm takes into account the primary and secondary structure information of proteins, fold-switching proteins whose secondary structure remains intact, but there are rearrangements in tertiary contacts and hydrogen bonding would likely not be picked up by our model.

## Supplementary Material

btaf635_Supplementary_Data

## Data Availability

The code used for fragment-picking analysis is publicly available on Zenodo (https://zenodo.org/records/14837336). The Zenedo repository also contains the proteome-level prediction data for the 57 proteome. The algorithm is also hosted within the web server (http://mbu.iisc.ac.in/∼anand/morpheus).
