# Supplementary material for: Morpheus: a fragment-based algorithm to predict fold-switching behaviour in proteins across proteomes
Source: Bioinformatics. 2025 Nov 24;41(12):btaf635. doi: 10.1093/bioinformatics/btaf635 (PMC12702140; doi:10.1093/bioinformatics/btaf635)
Supplement: btaf635_Supplementary_Data [file btaf635_supplementary_data.pdf]

Supplementary Information for

Morpheus: A Fragment-based Algorithm To Predict Fold-switching Behaviour In Proteins Across Proteomes

Vijay Subramanian[1,†], Rajeswari Appadurai[2,3,†], Harikrishnan Venkatesh[4], Ashok Sekhar[3], and Anand Srivastava[3,*]

[1]Indian Institute Of Science Education and Research, Pune 411008, India
[2]Department of Biology, Indian Institute of Science Education and Research, Tirupati 517619, India
[3]Molecular Biophysics Unit, Indian Institute of Science, Bangalore 560012, India
[4]International Business Machine (IBM), EGL Business Park, Bangalore 560071, India

[*]Corresponding author: anand@iisc.ac.in
[†]Equal contributions: Vijay Subramanian and Rajeswari Appadurai

Contents

1 Details of Materials and Methods **2**

1.1 Training Dataset 2

1.2 Database 2

1.3 Fragment Picking 2

1.4 Sequence Clustering 3

1.5 Filtering Morpheus Predictions 4

1.6 Scoring Metrics 6

1.6.1 Diversity and Entropy Scores 6

1.6.2 Substitution Score 6

1.6.3 Uncertainty 7

1.6.4 Hoeffding's Inequality 7

1.6.5 Final Score 7

1.6.6 Window width optimization 8

2 Additional Control Group: Intrinsically Disordered Proteins **8**

3 Supplementary Figures **9**

3.1 Figure S1: Schematic 9

3.2 Figure S2: Diversity Metrics 10

3.3 Figure S3: Cross-validation 16

3.4 Figure S4: Proteome Data 20

3.5 Figure S5: New Predictions 21

4 Dataset **26**

4.1 Fold-switching Proteins Dataset 26

4.2 Monomorphic Proteins Dataset 27

5 Optimizations **28**

1 Details of Materials and Methods

The methods used to perform sequence based fold-switching protein classification are explained in detail in the following sections.

1.1 Training Dataset

The training dataset has been borrowed from existing curated literature [1,2]. The set is further filtered to remove structures flagged as obsolete and structures that were later found not to be fold-switching due to erratum in structure. Refer to Section 4 for the PDB IDs of the entire training dataset of metamorphic proteins and proteins that are highly likely to be monomorphic.

1.2 Database

Fragment picking is performed on a curated database containing around 200,000 redundant proteins from the Protein Data Bank and around 1.2 million protein structure predictions from Alphafold2. This database is provided as is, along with the code for fragment picking online. Along with the sequence information, the database contains secondary structure and pLDDT score (if applicable) across each protein ID. As we perform the fragment search on a redundant PDB database, many of the hits occurring from different protein structures may all be pertaining to the same protein sequence. Hence, to eliminate the over-representation of proteins that have multiple redundant structures with the same sequence deposited in the PDB, we use a sequence clustering algorithm to cluster the database with a sequence identity threshold of 100%. Sequence clustering is performed using the fast and sensitive tool MMseqs2. All sequence alignments with sequence identity scores of 100% and covering at least 80% (-c 0.80) of both sequences are grouped in one cluster. Each of these clusters will have a representative cluster ID. This sequence cluster ID is also stored across each protein ID in the database. For the proteins from PDB that contain multiple chains, each chain's secondary structure information is extracted and stored separately in the database.

1.3 Fragment Picking

In order to perform fragment picking on a proteome, the fast lookup time of a trie data structure is leveraged. All the proteins in the proteome are broken into 7-mer fragments, and all the resulting 7-mers are stored in a trie. This trie now has all the possible fragments from the proteome that need to be searched for. So now, all we have to do is iterate once through the database and whenever any fragment from the trie is encountered, append all the associated structural information of that fragment in the leaf node of the trie corresponding to the same fragment. Fragment hits whose middle residue is an unobserved residue or has a local pLDDT score of less than 70 are removed in the case of experimentally solved structures and computationally solved structures, respectively

To perform trie-based fragment search, we have implemented the trie structure using the Python module 'pygtrie' developed by Google. Once an instance of the trie is created, the fragments from all the proteins in the proteome are stored one by one in the trie. Then, we scan the database for any of the fragments present in the trie with a linear

search. When we encounter any hit, the associated information is retrieved from the database and stored in the leaf node of the corresponding fragment in the trie. On our system (16-core AMD Ryzen 7 5700, 64 GB RAM), the trie-implemented search for a proteome with 20,000 proteins was completed within 2 hours.

For the web server, where fragment picking needs to be performed on a single user-defined protein sequence, the fragment search is performed by iterating through the lines of the database with a fast linear search in Java. This is done by matching 7-mer fragment sequences to the 7-mers in the database using a 'Regular expression' (REGEX) based search. When run on our system (16-core AMD Ryzen 7 5700, 64 GB RAM), the search for one fragment across the database is completed at an average of 700 milliseconds.

1.4 Sequence Clustering

All the sequences in the curated database are run through the sequence clustering tool MMSeqs2 and their cluster IDs are saved across each protein sequence in the database file. The sequence clustering was run with the conditions of 100% sequence identity and covering at least 80% (-c 0.80) of all the sequences in that cluster. When performing the fragment picking for a query sequence, multiple fragments may be obtained from different protein structures within the same cluster; to account for that, when secondary structure probabilities are calculated, each hit from a cluster is weighted in a manner that is displayed in Algorithm 1.

Algorithm 1 Pseudocode for Assigning Weights and Calculating Diversity Metrics

Require: A list of protein fragment 7-mers

Initialize $ss\_helix \leftarrow 0$, $ss\_sheet \leftarrow 0$, $ss\_coil \leftarrow 0$

for each fragment in the list **do**

  $middle\_ss \leftarrow$ secondary structure of the third residue

  $cluster\_id \leftarrow$ fragment sequence cluster ID

  $$weight \leftarrow \frac{1}{\text{total\_number\_of\_fragments\_in\_cluster}(cluster\_id)}$$

  if $middle\_ss = H$ **then** ▷ Helix

    $ss\_helix \leftarrow ss\_helix + weight$

  else if $middle\_ss = E$ **then** ▷ Sheet

    $ss\_sheet \leftarrow ss\_sheet + weight$

  else if $middle\_ss = L$ **then** ▷ Coil

    $ss\_coil \leftarrow ss\_coil + weight$

  end if

end for

$total\_prop \leftarrow ss\_helix + ss\_sheet + ss\_coil$

$p\_helix \leftarrow \frac{ss\_helix}{total\_prop}$

$p\_sheet \leftarrow \frac{ss\_sheet}{total\_prop}$

$p\_coil \leftarrow \frac{ss\_coil}{total\_prop}$

$diversity \leftarrow scorefn\_diversity(p\_helix, p\_sheet, p\_coil)$

$entropy \leftarrow scorefn\_entropy(p\_helix, p\_sheet, p\_coil)$

return Diversity and Entropy metrics for the fragment

1.5 Filtering Morpheus Predictions

If a protein sequence is predicted to be fold-switching by Morpheus on the basis of the 4 scoring metrics, we first demarcate the protein sequence into a fold-switching region and a non-fold-switching region. This is done by using a threshold information entropy score, residues which have an entropy score higher than (or equal to) the threshold are categorized as belonging to a fold-switching region. To achieve this, we first need to arrive at a threshold information entropy score in a rational method.

This is done by analysing the entropy scores in the fold-switching region (FS region) of the training dataset of fold-switching proteins. These fold-switching regions are already known through experiments, and hence we can tailor our threshold entropy score so that it is able to find these regions correctly. To do this, we sample a range of entropy thresholds from [0,ln(3)] and calculate the following scores: positive score and negative score.

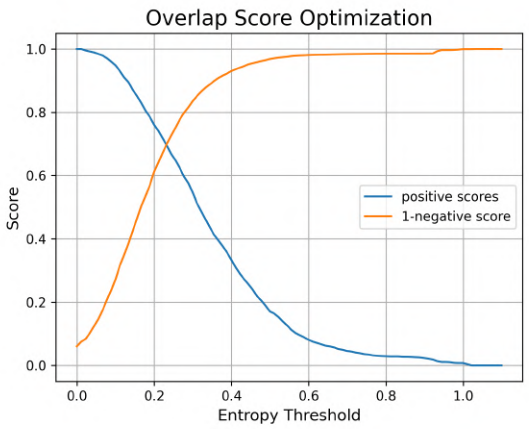

Figure 1: The variation of positive score and (1 - negative score) with different entropy thresholds. The objective is to find a threshold which has the maximum positive and (1-negative) score simultaneously. We chose the information entropy score corresponding to the intersection of the two functions plotted (Entropy = 0.231) as the threshold entropy score

$$\text{positive score} = avg\left(\frac{\text{\# of residues correctly marked in the FS region}}{\text{total \# of residues in the FS region}}\right)$$

$$\text{negative score} = avg\left(\frac{\text{\# of residues incorrectly marked in the non-FS region}}{\text{total \# of residues in the non-FS region}}\right)$$

In the above expressions, the Avg represents the average of these scores across the entire training fold-switching dataset. Our objective is to find an entropy threshold that maximizes positive score while maintaining a minimum negative score.
Based on Figure 1, we chose the information entropy score corresponding to the intersection of the two functions plotted (Entropy = 0.231) as the threshold entropy score.

Next, we run the protein sequence through two disorder predicting tools: IUPred2A and Metapredict. We take a consensus of the regions predicted to be disordered by both the tools. Next we check if more than 50% of the region predicted fold-switching by Morpheus has overlap with the disorder consensus, then we do not consider these protein sequences in the final prediction set.

Further, we leverage the Pfam database of protein families and domains to check if the region predicted to be fold-switching belongs to a protein domain. Pfam is a database of protein families that includes their annotations and multiple sequence alignments generated using hidden Markov models. UniProt server has compiled the Pfam domains in their website for each protein within the UniProtKB database, we used the python requests library to scrape the protein domain information.

1.6 Scoring Metrics

1.6.1 Diversity and Entropy Scores

Once the identical fragments are picked from our database, the diversity and entropy scores are calculated using the propensity of the 3rd residue to take helix, sheet or loop secondary structure. The propensity of the 3rd residue is estimated from the outputs of fragment picking using classical probability. When performing the fragment picking for a query sequence, multiple fragments may be obtained from different protein structures within the same cluster; to account for that, when secondary structure probabilities are calculated, each hit from a cluster is weighted by the inverse of the total number of hits from the same cluster. After this weighted calculation of secondary structure propensities, we use them to score the particular fragment using diversity and entropy scores.

The Diversity and entropy scores are defined as follows:

$$\mathrm{DI} = (h^2 + e^2 + l^2)^{-1}$$
$$\mathrm{Entropy} = -h\log(h) - e\log(e) - llog(l)$$

1.6.2 Substitution Score

In bioinformatics, substitution matrix-based scoring techniques are widely used. They are particularly helpful in quantifying the change or substitution of one element by another. We implemented a substitution matrix based scoring method in order to evaluate the ability of the fragments to adopt different secondary structure conformation.
First, the secondary structure of the fragments in the original output from DSSP is taken. The original output from DSSP contains one of these 8 secondary structures: α-helix (H), 3_{10} helix (G), π helix (I), poly-proline helix (P), extended strand (E), β bridge (B), turn (T), bend (S). A reference hit is set as the hit that has the most commonly occurring 7-mer secondary structure pattern among all the hits. If there is no majority, a random hit is assigned as the reference hit. Then every other hit is taken, pair-wise aligned with the reference hit and scored according to the following substitution matrix:

$$\frac{1}{7}\begin{bmatrix} \text{ref/replace} & \text{H/I/G} & \text{E/B} & \text{T} \\ \text{H/I/G} & 0 & 0.7 & 0.3 \\ \text{E/B} & 0.7 & 0 & 1 \\ \text{T} & 0.3 & 1 & 0 \end{bmatrix}$$

This way, as we align each fragment sequence hit with the reference hit and apply the substitution matrix on the alignment, we get a score. For a sliding window fragment, all pairwise alignment with the reference hit is taken, and among them, if there are multiple hits from the same sequence cluster, these scores are weighted with the inverse of the total number of hits that we obtain from the same sequence cluster. We then take the weighted average of these scores from all pairwise alignments and assign it to that sliding window fragment. In this manner, we obtain a score for each fragment corresponding to all sliding windows.

1.6.3 Uncertainty

Ideally, there exists a true value of the propensity for the middle residue to be in a helical or sheet-like structure; the propensity that we arrive at is an estimate that gets better and better based on the number of fragment hits from different sequence clusters that we have available to us to make an estimation. So we sought to quantify the confidence in the calculated diversity metrics for each sliding window based on the number of hits per fragment. Using Hoeffding's inequality (Refer Section 1.6.4), we have modelled the uncertainty as an exponentially decreasing function of the sample size as shown in the equation that follows.

$$\text{Uncertainty} = \exp(-0.5 \times \#\text{ of hits from unique clusters})$$

The number of hits from unique clusters obtained through fragment picking is computed under the assumption that the query protein sequence is itself a member of a cluster; accordingly, one cluster is subtracted from the total count of unique clusters.

1.6.4 Hoeffding's Inequality

Hoeffding's inequality is a statistical bound that quantifies how rapidly the empirical frequency of an event converges to its true probability as the number of independent samples increases. For a sample size of N, the relation is given by the following equation:

$$\mathbb{P}[|\nu - \mu| < \epsilon] \leq 2e^{-2\epsilon^2 N} \text{ for any } \epsilon > 0$$

Here, ν represents the estimated probability of an event when it is sampled N times, and μ represents the true probability of the event. Thus the probability of a deviation at least ϵ decays exponentially in the sample size N at rate $2\epsilon^2$.

1.6.5 Final Score

The final score that is used as the input vector for the classification model is the four aforementioned scores together. The four scores together are used as the feature space for the classification model, as shown below:

$$\text{Final Score} = \begin{pmatrix} max\left(\frac{1}{WW}\sum_{j=0}^{j<WW} \text{Diversity}_{i+j}\right) \\ max\left(\frac{1}{WW}\sum_{j=0}^{j<WW} \text{Entropy}_{i+j}\right) \\ max\left(\frac{1}{WW}\sum_{j=0}^{j<WW} \text{Substitution}_{i+j}\right) \\ mean(\text{uncertainty}) \end{pmatrix}$$

If no hits are found for a particular fragment, we assign the minimum scores for: diversity = 1, entropy = 0, substitution = 0, and maximum score for uncertainty = 1.0. This is done to minimize false positives when we have no data to estimate the diversity of the fragment.

1.6.6 *Window width optimization*

We theorized that a protein sequence having a contiguous region of high entropy scores would imply that the region as a whole would have a higher chance of switching folds. Hence, we chose to make a rolling average of the scores to elicit these contiguous regions. The rolling window width is a parameter that we are at liberty to choose. It is found that a window width of 8 and 18 gives the best separation of metamorphic and monomorphic scores in the training data (Refer to Table S3). Hence, a step function, as shown below, is used for the rolling average window:

$$\text{Window width (WW)} = \begin{cases} 8 & \text{len(protein sequence)} \leq 25 \\ 18 & \text{len(protein sequence)} > 25 \end{cases}$$

2 Additional Control Group: Intrinsically Disordered Proteins

Proteins that are known to be intrinsically disordered proteins are run through Morpheus' fragment picking algorithm. The additional control groups used are intrinsically disordered proteins that are known to conditionally fold, and those intrinsically disordered proteins that have a very high disordered state. The conditional folding intrinsically disordered proteins dataset was obtained from DisProt with tag 'disorder to order structural transitions' (IDPO:00050), and intrinsically disordered proteins with no conditional folding dataset was obtained from DisProt with tag 'flexible linker/spacer' (IDPO:00502). Figure 2 shows the distribution of diversity metrics for these two additional control groups. Referring to Figure 2 we can see that the diversity metrics for the two additional control groups are distributed in both the predicted monomorphic and fold-switching region of the phase space. Hence when making new predictions, additional filtering steps detailed in section 1.5 are needed to filter out the intrinsically disordered proteins.

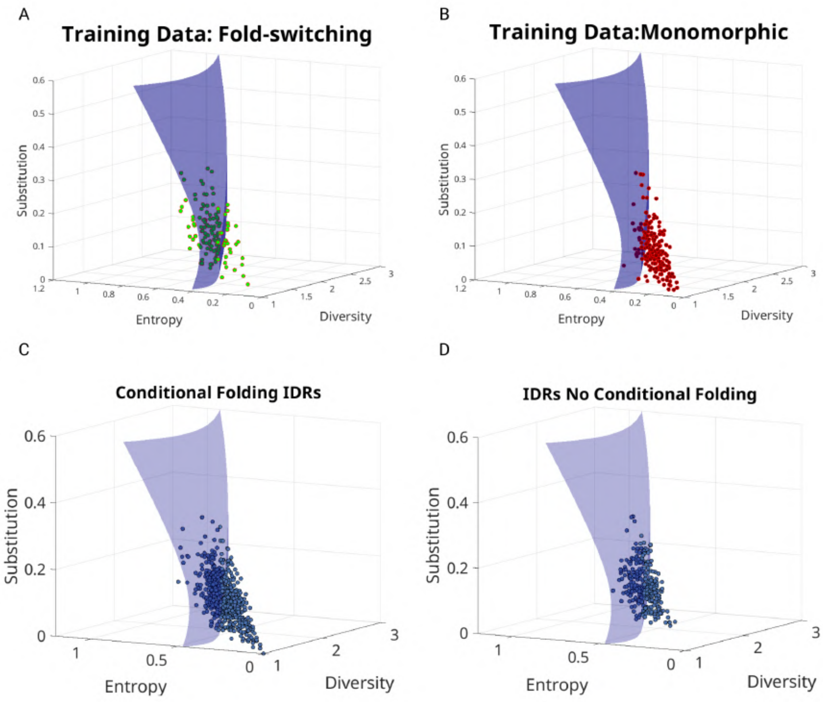

Figure 2: **Control Groups' Distribution**:protein sequences plotted in the 3-dimensional feature space of diversity, entropy and substitution score for A) Training Fold-switching, B) Training Monomorphic, C) Conditionally Folding IDR protein sequences and D) IDRs whose function depend on their high disorder (no presumed conditional folding). The conditional folding IDR dataset was obtained from DisProt with tag 'disorder to order structural transitions' (IDPO:00050), and IDRs with no conditional folding dataset was obtained from DisProt with tag ' flexible linker/spacer' (IDPO:00502).

3 Supplementary Figures

3.1 Figure S1: Schematic

Figure S1.a shows the fragment picking schematic with the cartoon representation of the fragments from different hits and their respective PDB ID/AlphaFold Accession numbers.

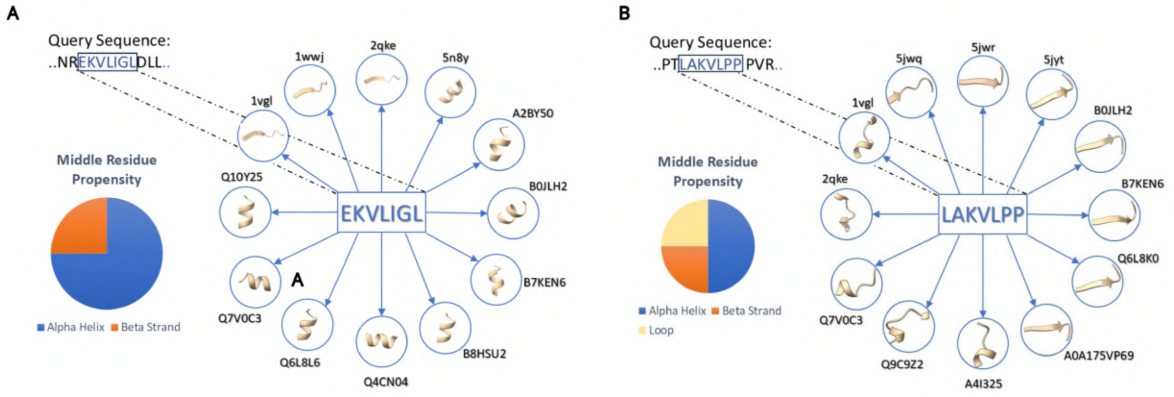

Figure S1.a: Schematic for fragment picking. The fragment sequences shown are part of the metamorphic protein KaiB **A** EKVLIGL and **B** LAKVLPP

3.2 Figure S2: Diversity Metrics

The 10 figures in S2 show the diversity metrics (diversity, information entropy, substitution score and uncertainty) for 10 different experimentally known metamorphic proteins. They are RfaH, KaiB, IscU, Mad2, Lymphotactin, Selecase, MinE, CLIC1, HIV-RT1, and the designed metamorphic protein. The plots contain calculated helix and sheet propensities, the entropy scores, the diversity scores and Substitution scores at each sliding window. The uncertainty score at each fragment is also taken into a rolling average and overlayed on the diversity score. The experimentally solved structures for the two conformations are also displayed.

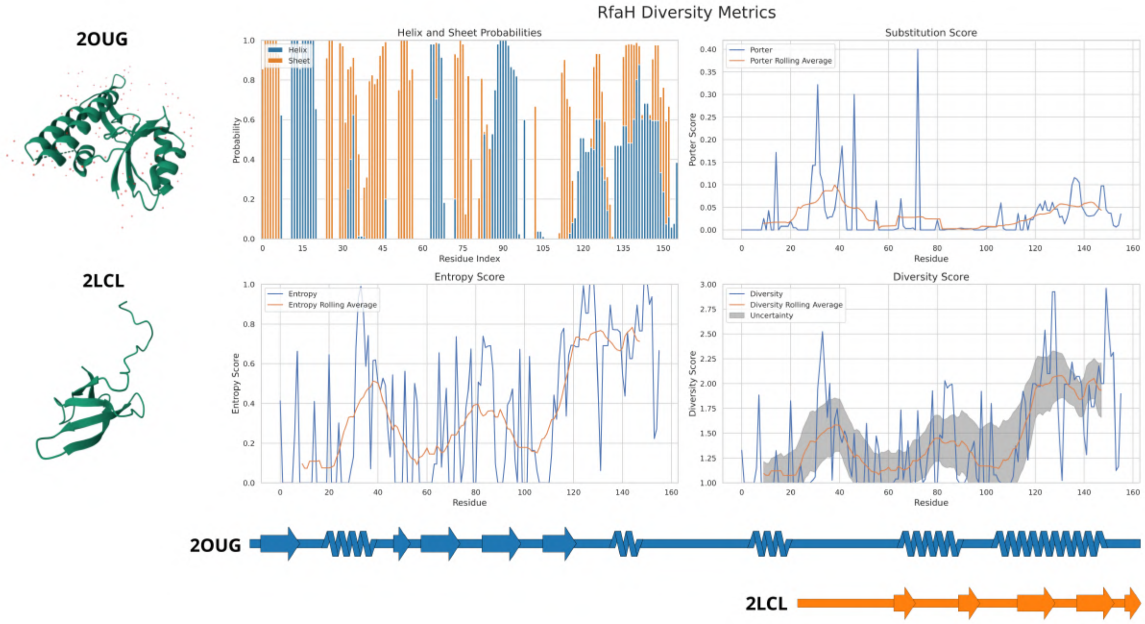

Figure S2.a: **RfaH:** Diversity metrics and secondary structure information for the metamorphic protein RfaH. The corresponding PDB IDs are 2OUG and 2LCL.

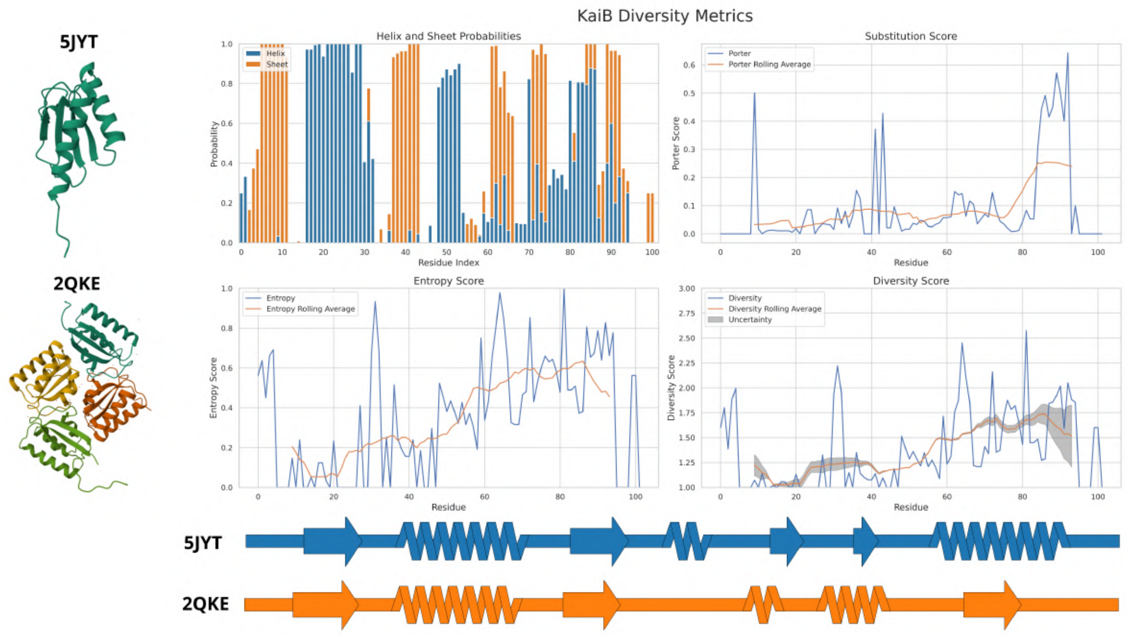

Figure S2.b: **KaiB:** Diversity metrics and secondary structure information for the metamorphic protein KaiB. The corresponding PDB IDs are 5JYT and 2QKE.

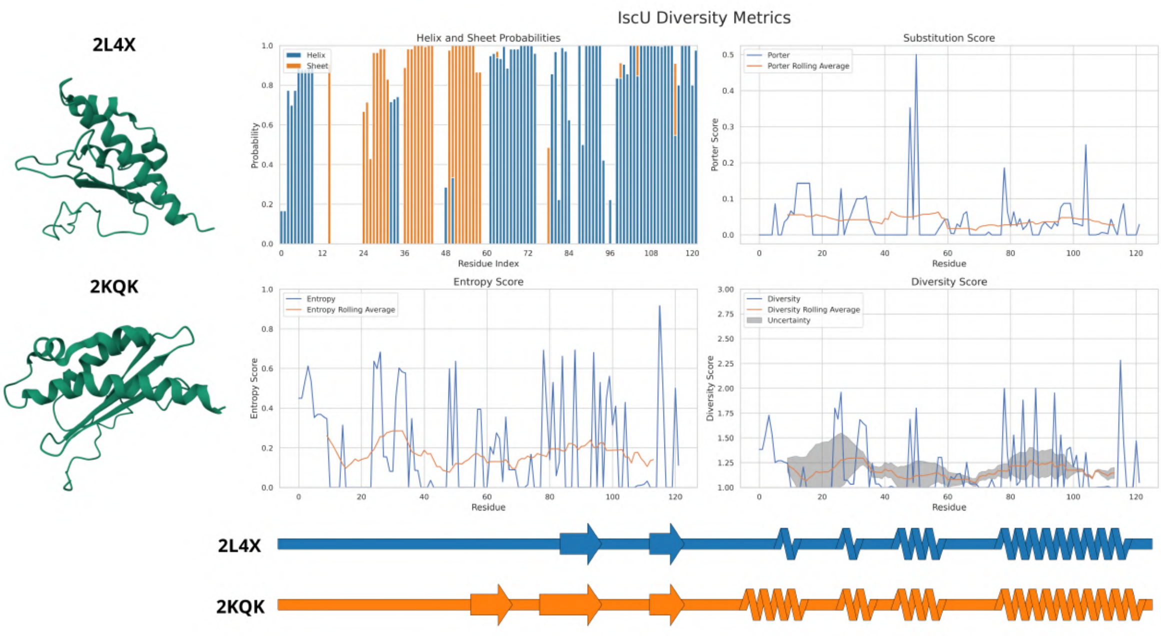

Figure S2.c: **IscU:** Diversity metrics and secondary structure information for the metamorphic protein IscU. The corresponding PDB IDs are 2L4X and 2KQK.

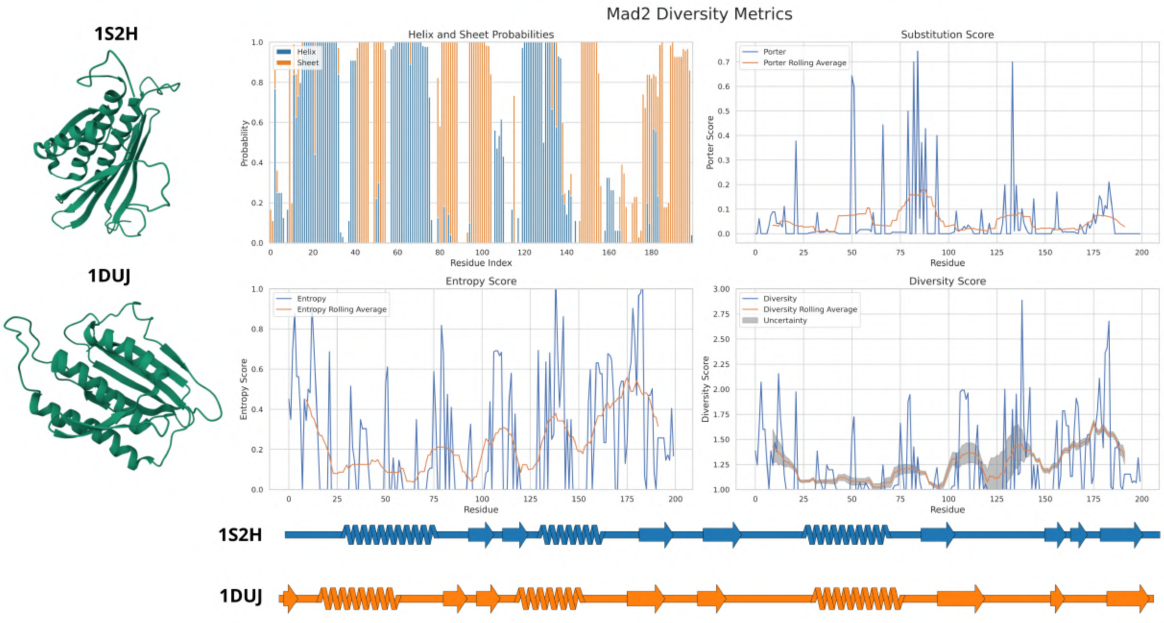

Figure S2.d: **MAD2:** Diversity metrics and secondary structure information for the metamorphic protein MAD2. The corresponding PDB IDs are 1S2H and 1DUJ.

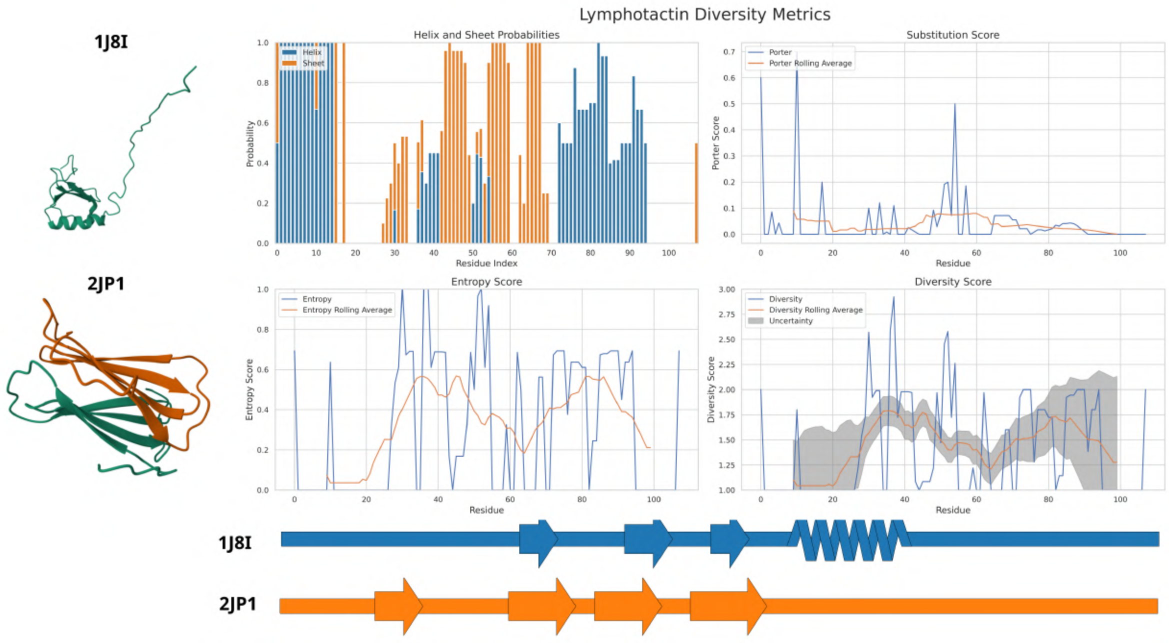

Figure S2.e: **Lymphotactin:** Diversity metrics and secondary structure information for the metamorphic protein Lymphotactin. The corresponding PDB IDs are 1J8I and 2JP1.

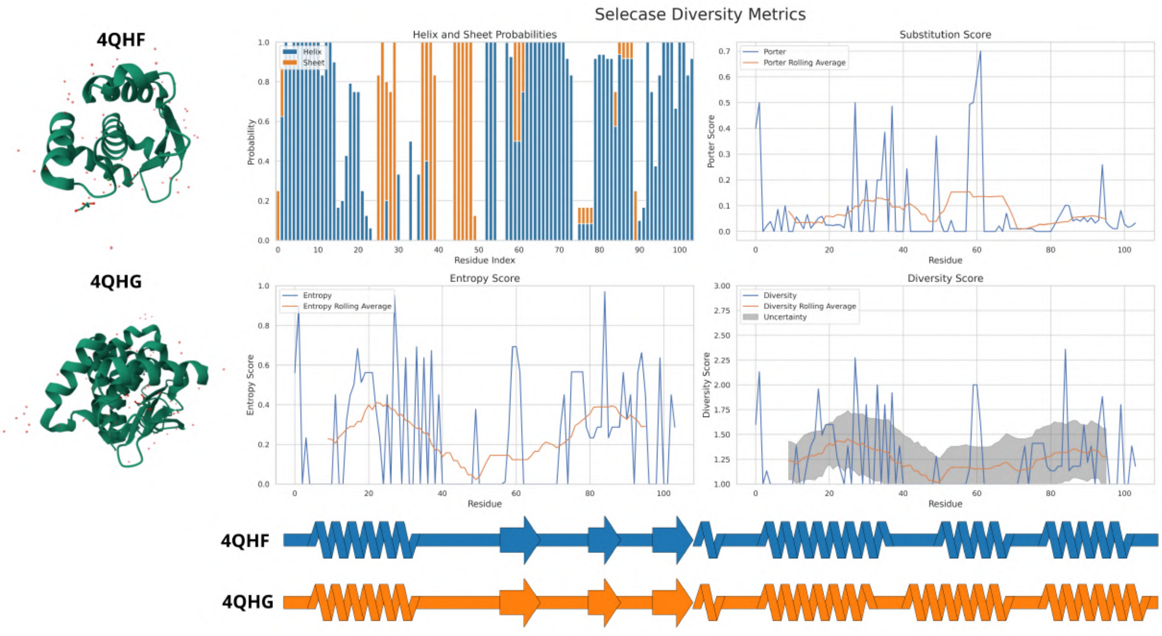

Figure S2.f: **Selecase:** Diversity metrics and secondary structure information for the metamorphic protein Selecase. The corresponding PDB IDs are 4QHF and 4QHG.

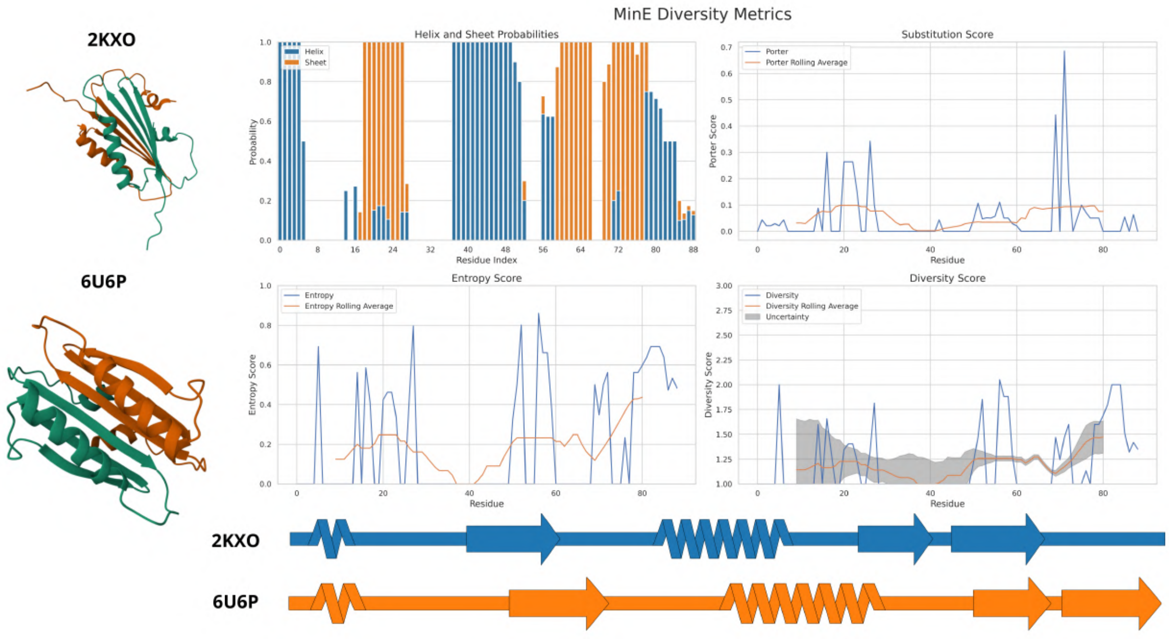

Figure S2.g: **MinE:** Diversity metrics and secondary structure information for the metamorphic protein MinE. The corresponding PDB IDs are 2KXO and 6U6P.

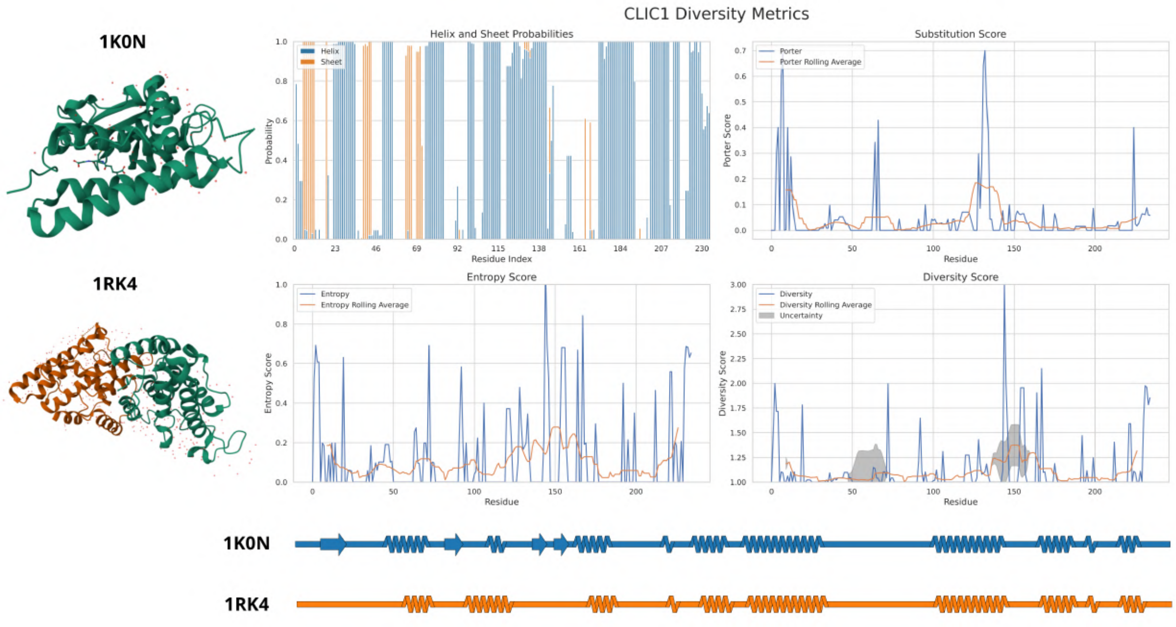

Figure S2.h: **CLIC1:** Diversity metrics and secondary structure information for the metamorphic protein CLIC1. The corresponding PDB IDs are 1K0N and 1RK4.

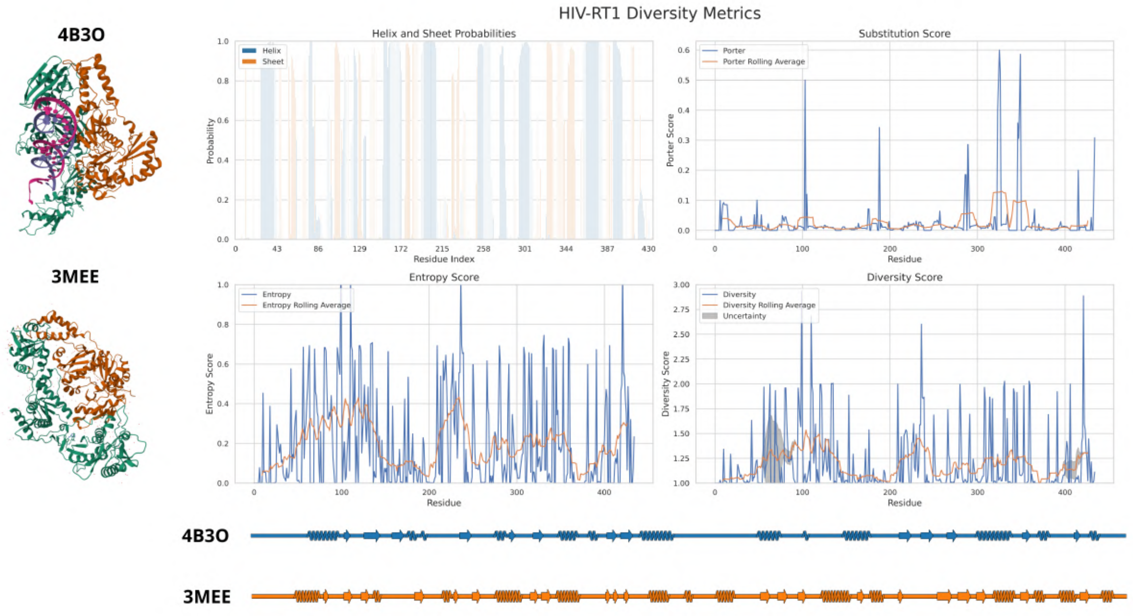

Figure S2.i: **HIV-RT1:** Diversity metrics and secondary structure information for the metamorphic protein HIV Reverse Transcriptase 1. The corresponding PDB IDs are 4B3O and 3MEE.

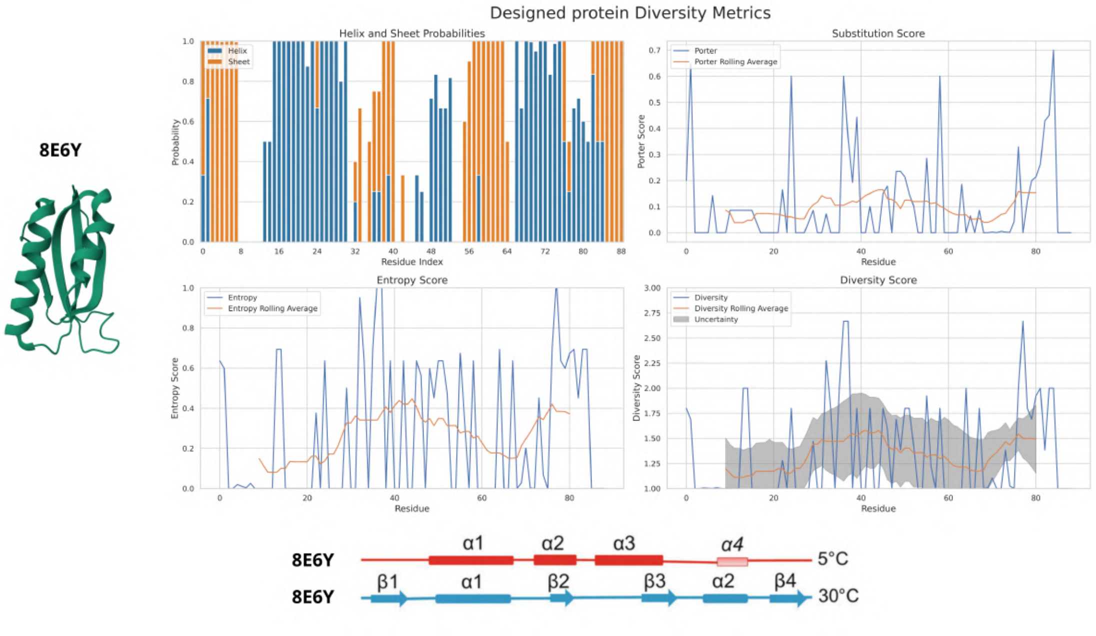

Figure S2.j: **Designed Protein:** Diversity metrics and secondary structure information for designed metamorphic protein. The corresponding PDB ID is 8E6Y.

3.3 *Figure S3: Cross-validation*

Figures S3 show how the decision boundary changes when the quadratic SVM model is trained with the 6 different splits of 6 fold cross-validation. The corresponding validation confusion matrix is also shown. Figure S3.f shows the ROC curve and aggregate confusion matrix (all 6 folds) for the final trained SVM model. S3.h shows the parallel coordinate plot and χ^2 scores from feature selection test for the four diversity metrics.

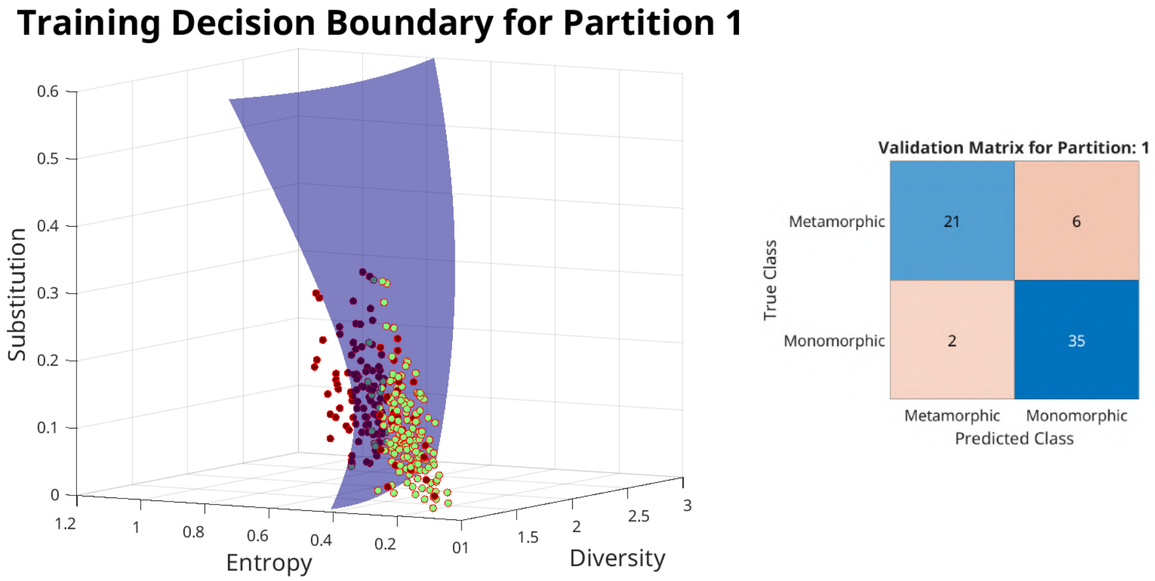

Figure S3.a: Decision boundary and the corresponding validation confusion matrix for the first training split partition

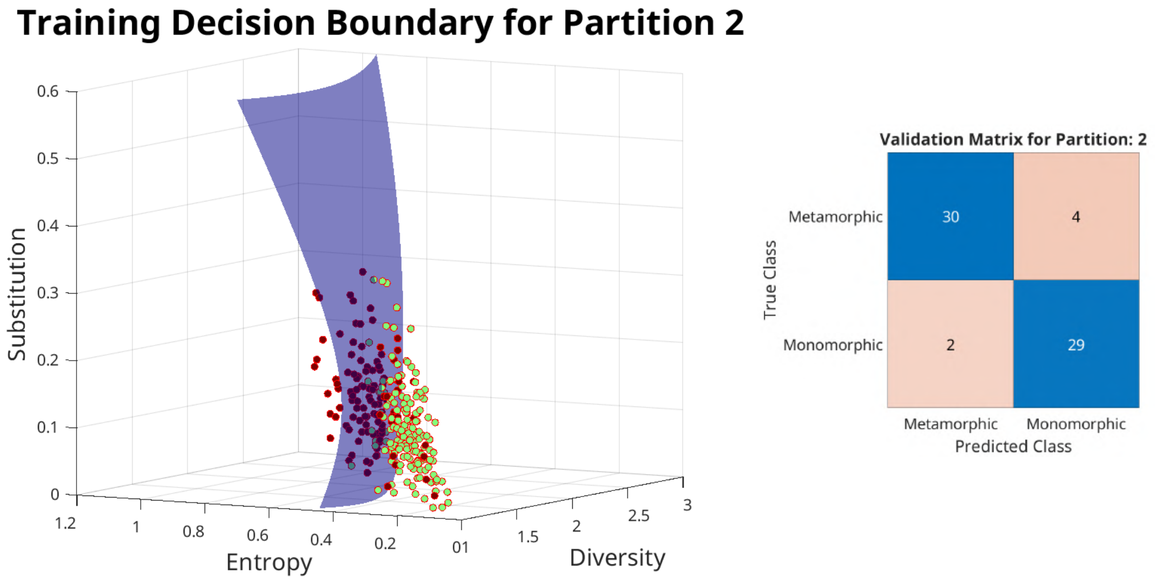

Figure S3.b: Decision boundary and the corresponding validation confusion matrix for the second training split partition

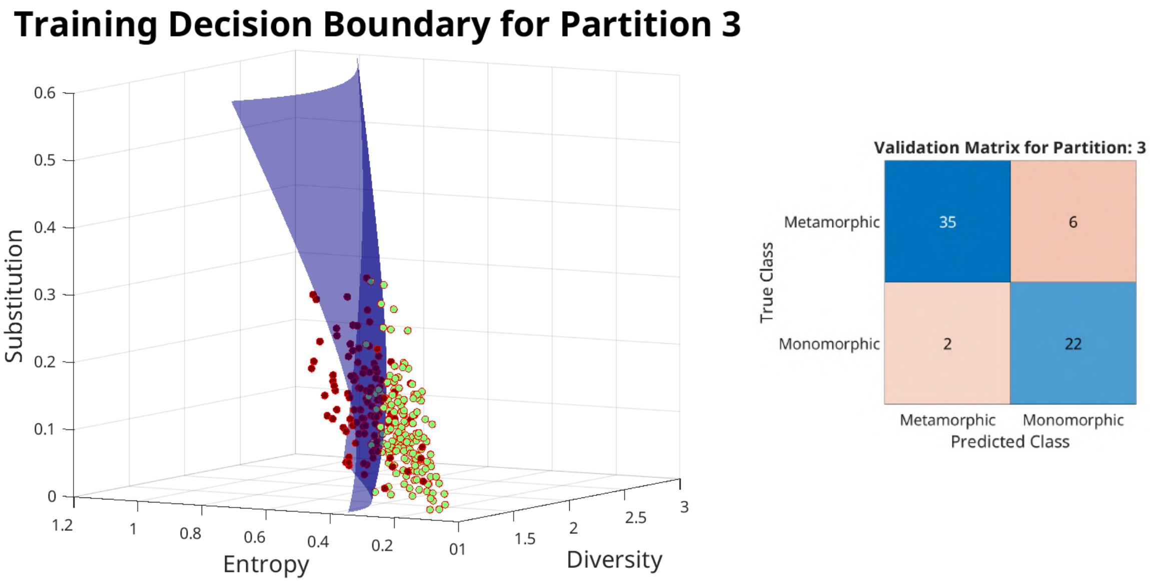

Figure S3.c: Decision boundary and the corresponding validation confusion matrix for the third training split partition

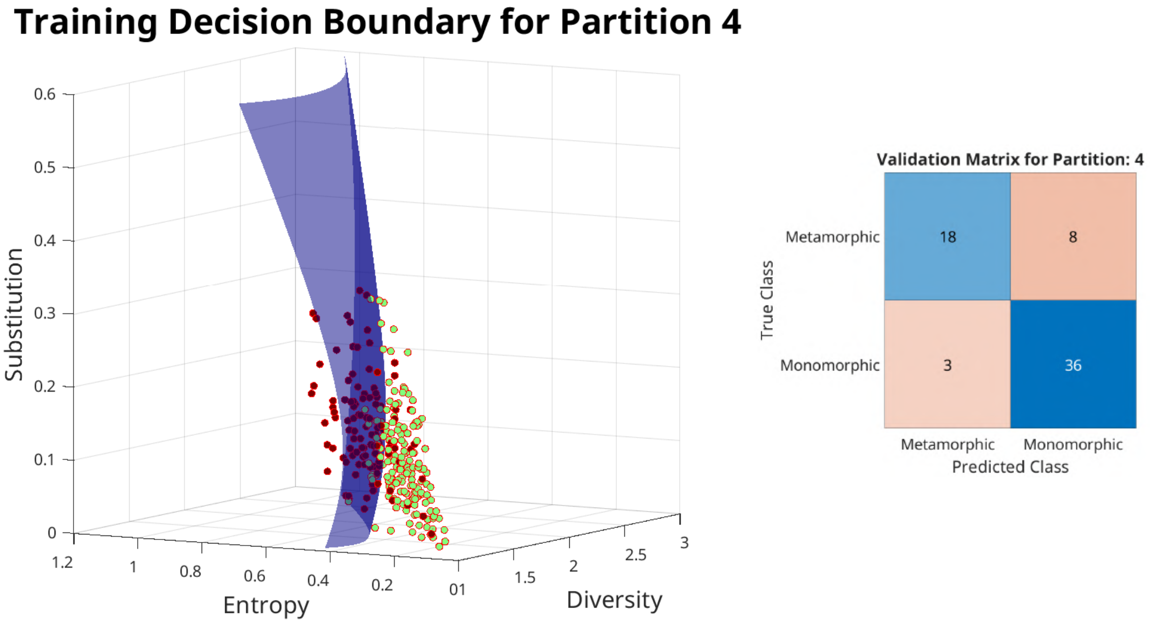

Figure S3.d: Decision boundary and the corresponding validation confusion matrix for the fourth training split partition

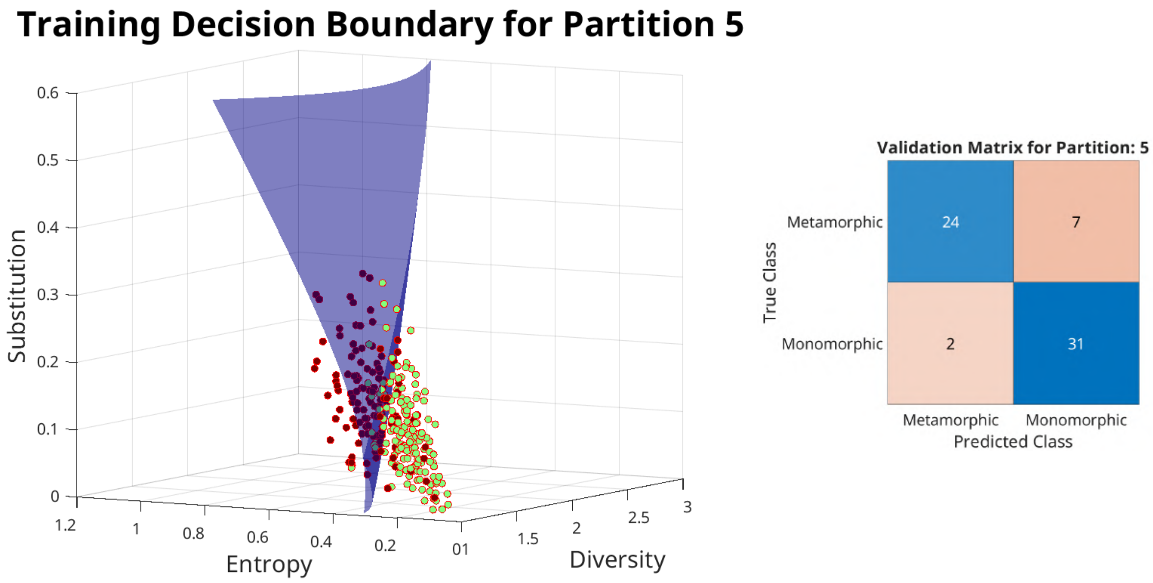

Figure S3.e: Decision boundary and the corresponding validation confusion matrix for the fifth training split partition

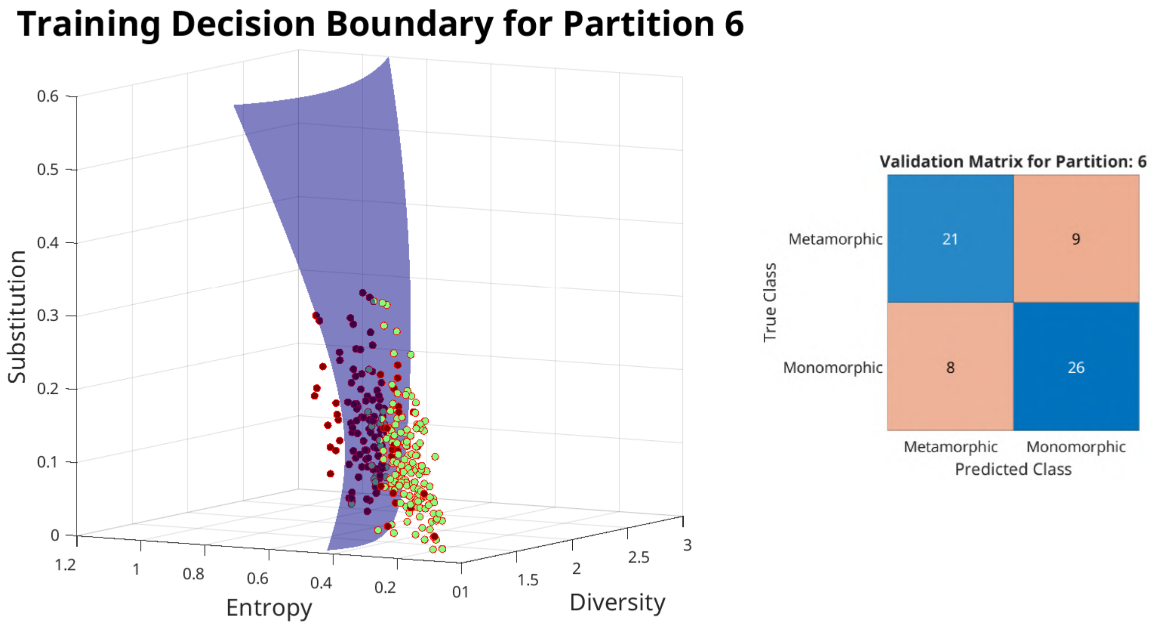

Figure S3.f: Decision boundary and the corresponding validation confusion matrix for the sixth training split partition

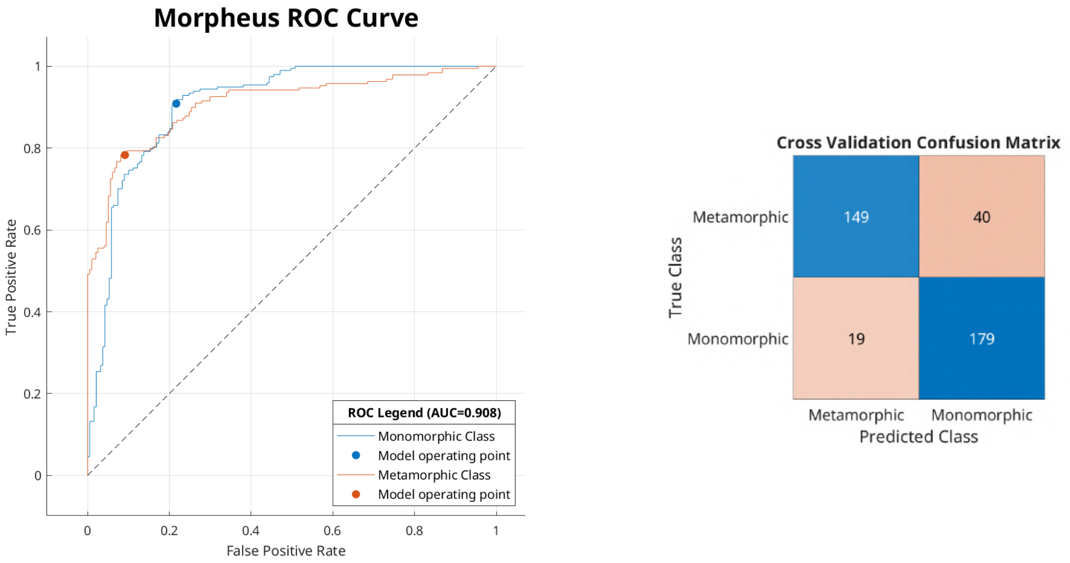

Figure S3.g: The Receiver-operating characteristic (ROC) curve for the final quadratic SVM classifier model and the final cross-validation matrix. The ROC curve is plotted considering the metamorphic protein class as the positive class. The area under the curve (AUC) for ROC curve is 0.89.

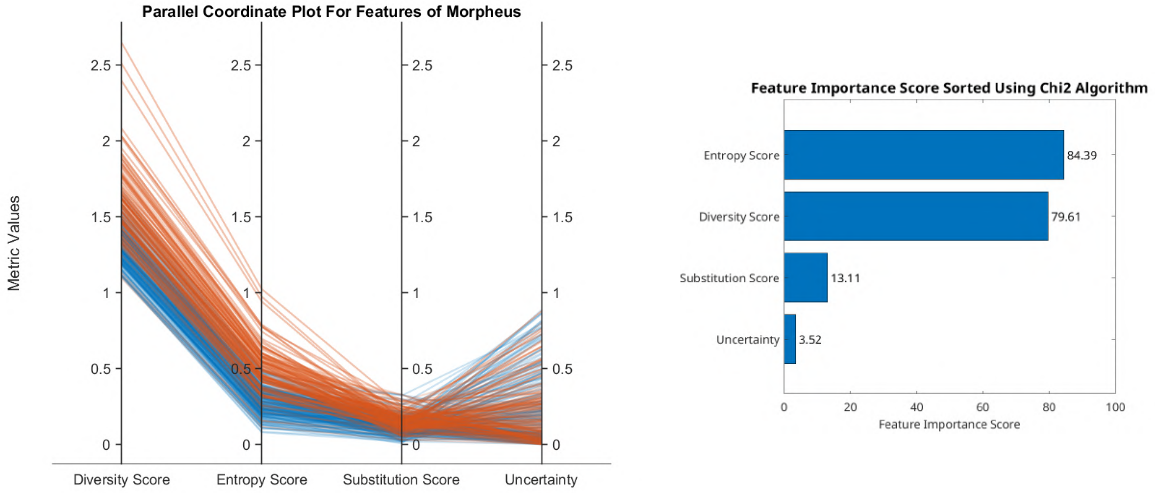

Figure S3.h: The parallel coordinate plot and χ^2 scores for the four features diversity, entropy, substitution score and uncertainty. The lines plotted in orange represent metamorphic proteins, and the lines in blue represent monomorphic proteins in the training dataset. The features are not transformed while plotting but are shown as is.

3.4 *Figure S4: Proteome Data*

Figures S4 shows the proteome data plotted on the feature space of diversity, entropy and substitution scores. The region to the left of the decision boundary represents the proteins that are predicted to be metamorphic, and the regions on the right are the ones predicted to be monomorphic.

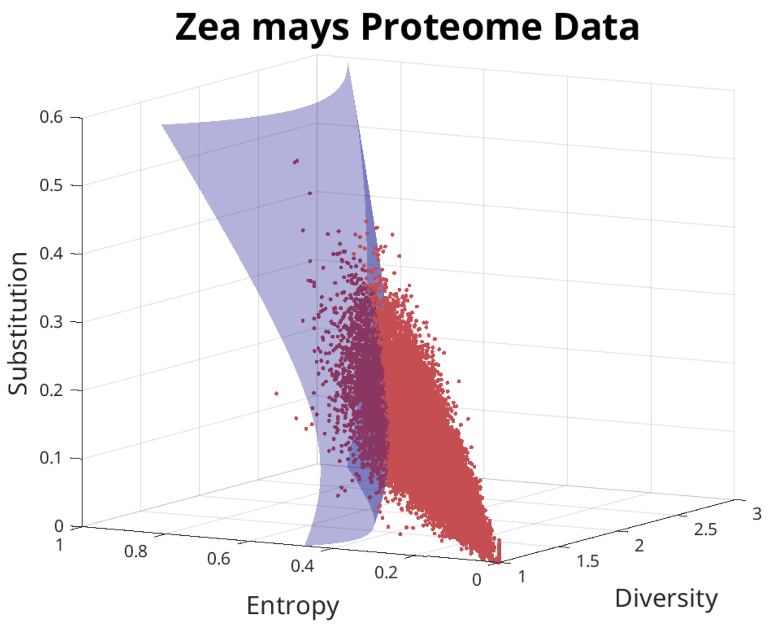

Figure S4.a: **Maize** proteome data plotted in the feature space of Diversity score, entropy score and substitution score. The surface represents the decision boundary obtained by the quadratic SVM model.

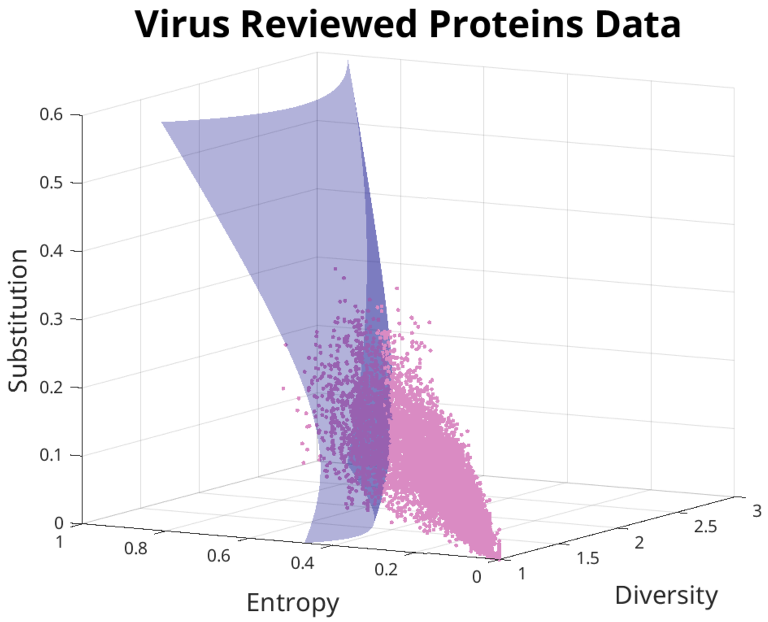

Figure S4.b: **Manually reviewed proteins that belong to the viral proteomes** are plotted in the feature space of Diversity score, entropy score and substitution score. The surface represents the decision boundary obtained by the quadratic SVM model.

3.5 Figure S5: New Predictions

In the set of figures S5, the diversity metrics are plotted for the selected few predictions listed in the paper. The plots contain calculated helix and sheet propensities, the entropy scores, the diversity scores and Substitution scores at each sliding window. The uncertainty score at each fragment is also taken into a rolling average and overlayed on the diversity score.

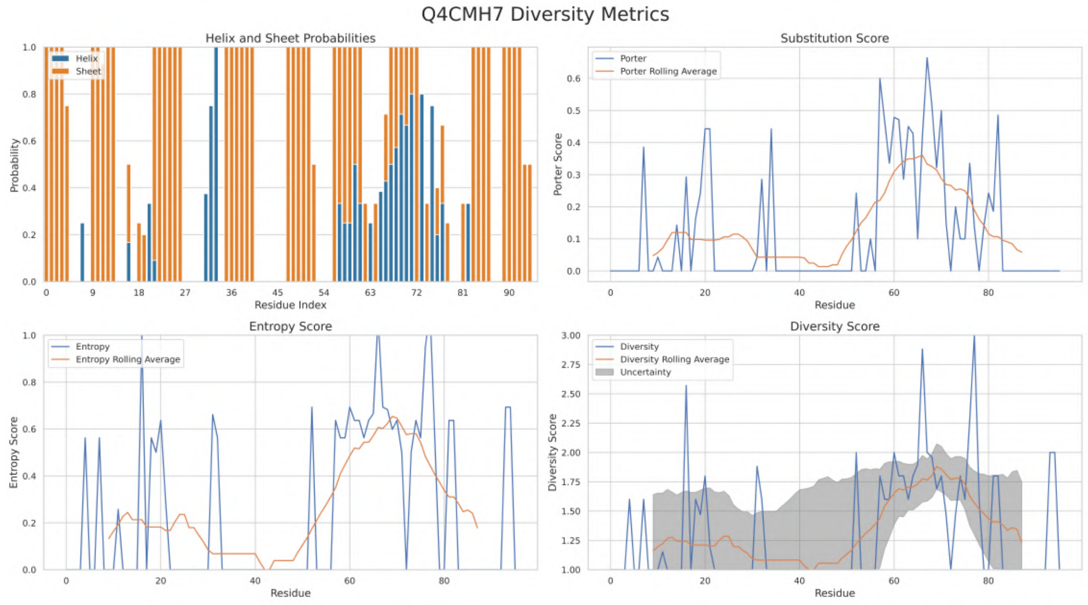

Figure S4.a: **Guanine nucleotide-binding protein subunit beta-like protein:** The diversity metrics plotted for the protein "Guanine nucleotide-binding protein subunit beta-like protein". Entropy, Diversity, Substitution and uncertainty are plotted above.

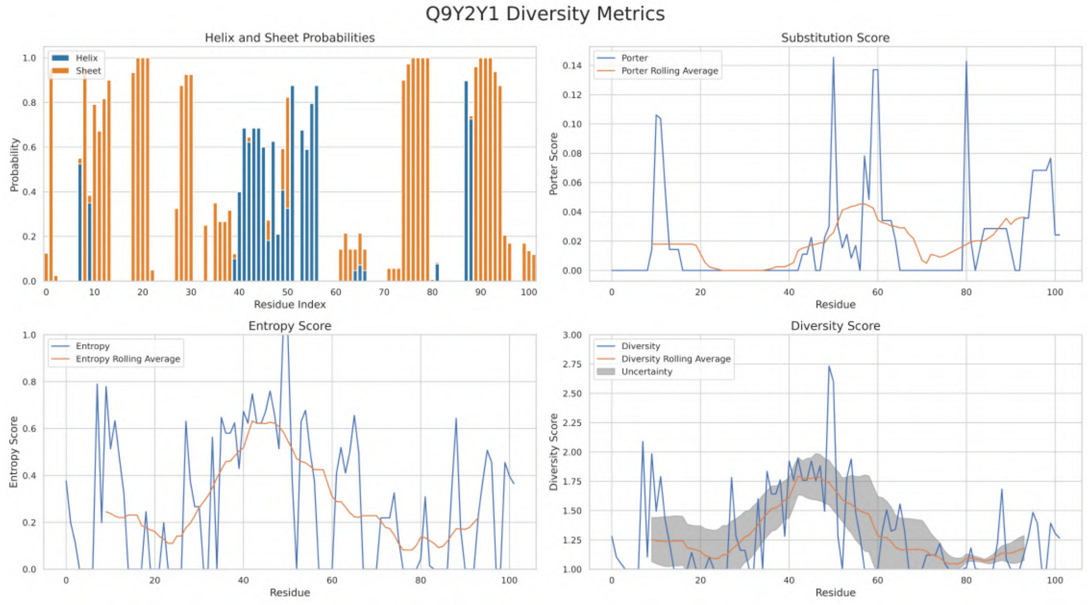

Figure S4.b: **DNA-directed RNA polymerase III subunit RPC10:** The diversity metrics plotted for the protein "DNA-directed RNA polymerase III subunit RPC10". Entropy, Diversity, Substitution and uncertainty are plotted above.

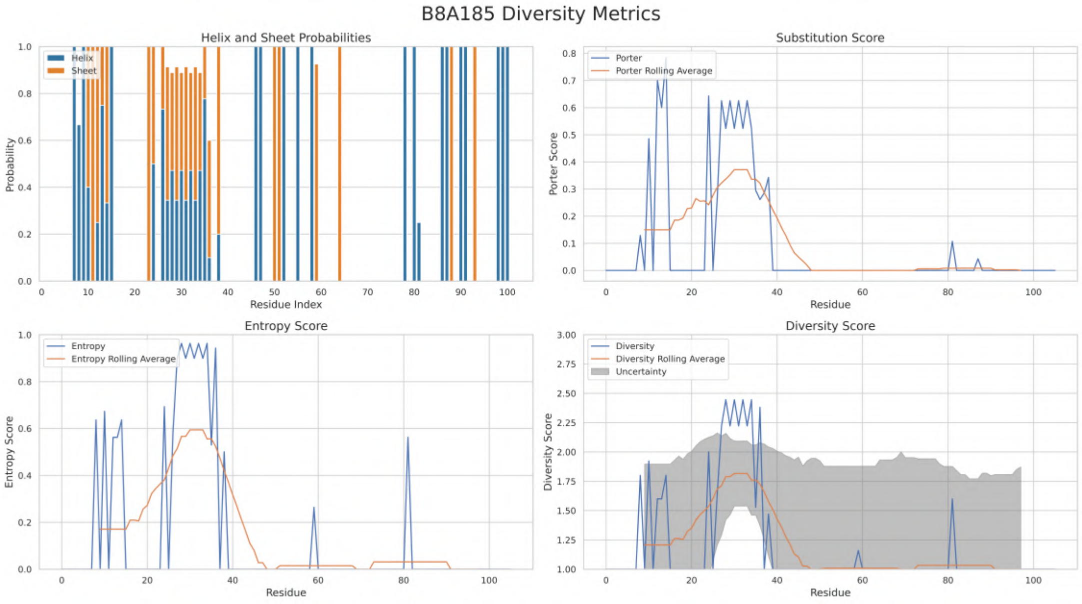

Figure S4.c: **Secreted protein:** The diversity metrics plotted for the Secreted protein. Entropy, Diversity, Substitution and uncertainty are plotted above.

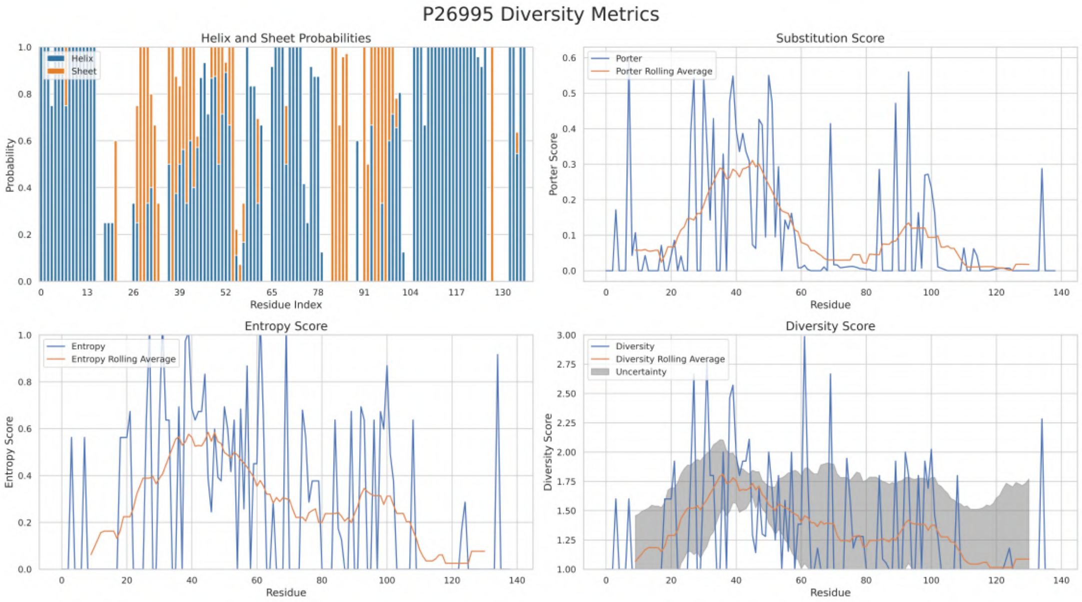

Figure S4.d: **Transcriptional anti-antiactivator ExsC:** The diversity metrics plotted for the protein "Transcriptional anti-antiactivator ExsC". Entropy, Diversity, Substitution and uncertainty are plotted above.

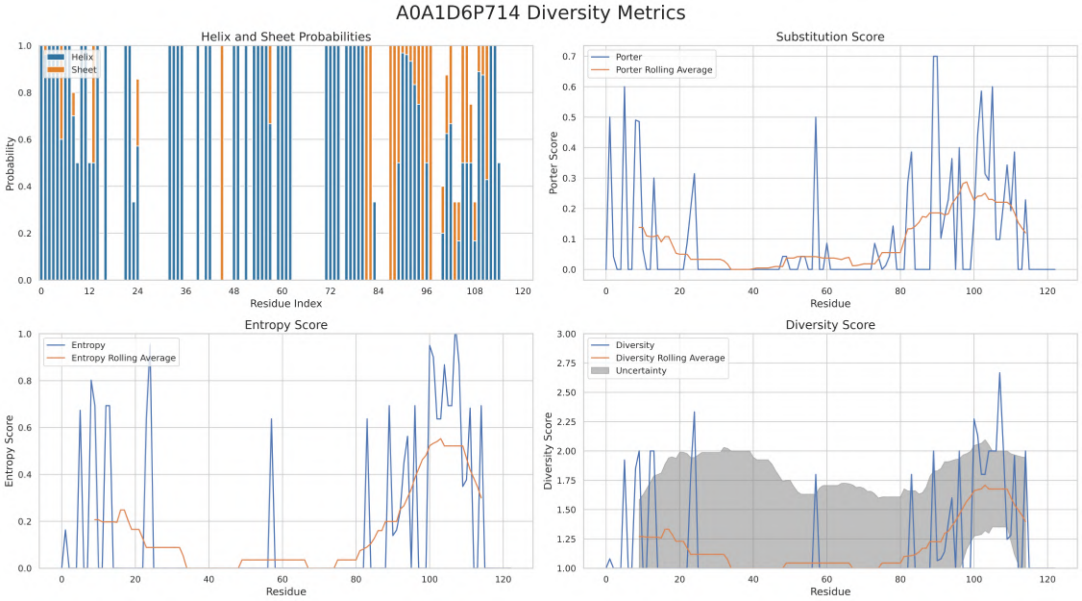

Figure S4.e: **Extensin-like protein:** The diversity metrics plotted for the protein "Extensin-like protein". Entropy, Diversity, Substitution and uncertainty are plotted above.

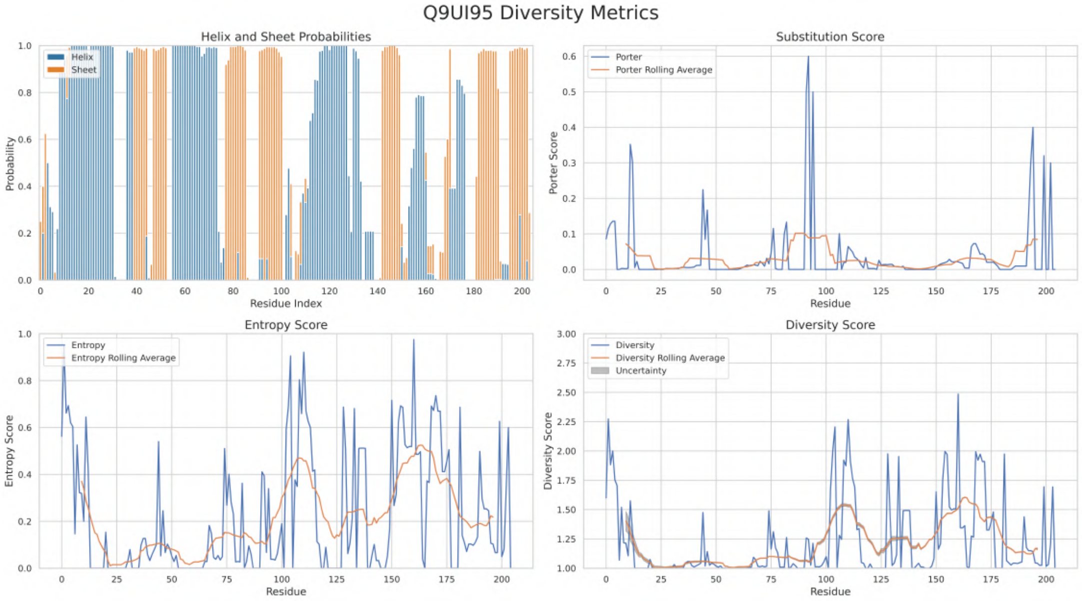

Figure S4.f: **Mitotic spindle assembly checkpoint protein MAD2B:** The diversity metrics plotted for the protein "Mitotic spindle assembly checkpoint protein MAD2B". Entropy, Diversity, Substitution and uncertainty are plotted above.

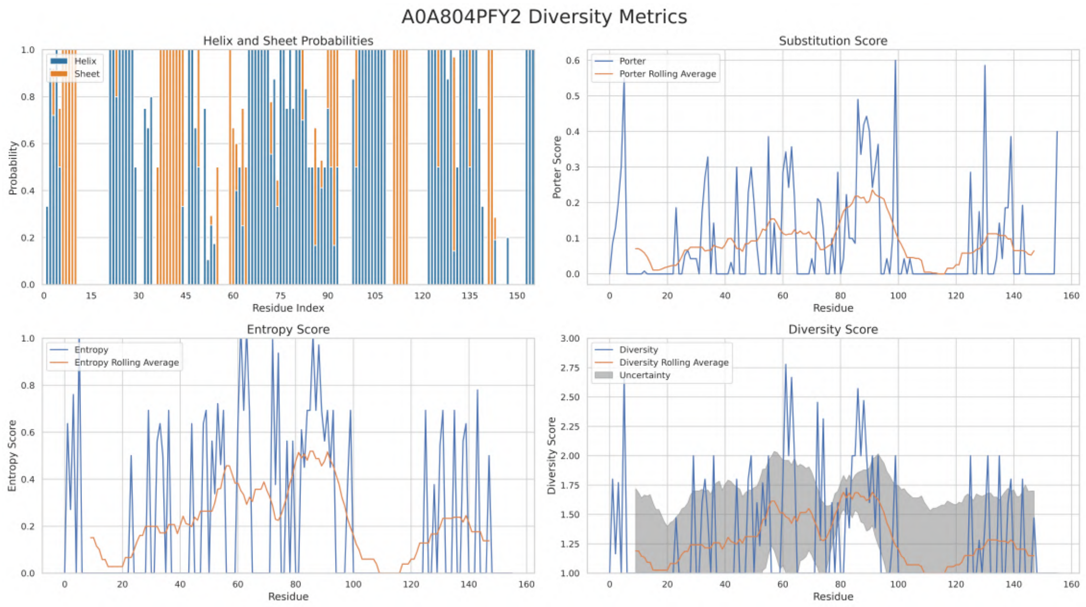

Figure S4.g: **Photolyase/cryptochrome alpha/beta domain-containing protein:** The diversity metrics plotted for the protein "Photolyase/cryptochrome alpha/beta domain-containing protein". Entropy, Diversity, Substitution and uncertainty are plotted above.

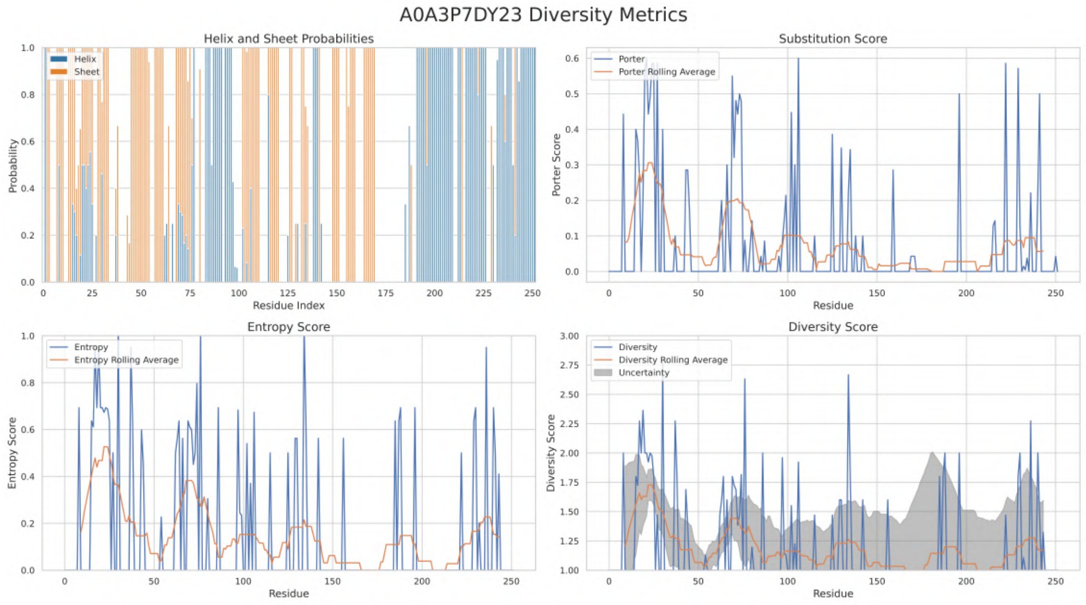

Figure S4.h: **S1 motif domain-containing protein:** The diversity metrics plotted for the protein "S1 motif domain-containing protein". Entropy, Diversity, Substitution and uncertainty are plotted above.

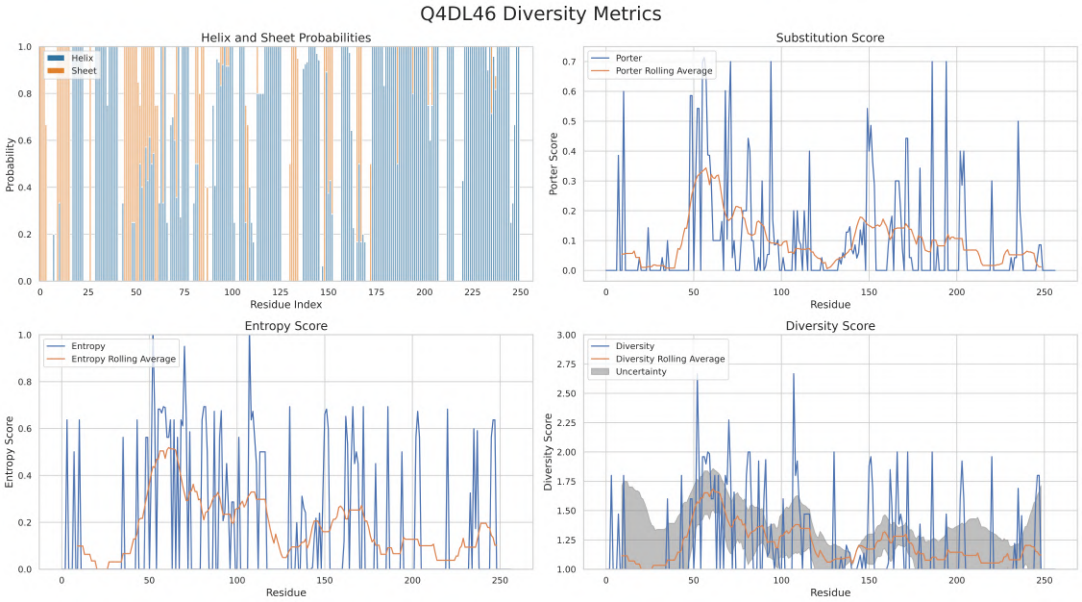

Figure S4.i: **Copper-transporting ATPase-like protein, putative:** The diversity metrics plotted for the protein "Copper-transporting ATPase-like protein, putative". Entropy, Diversity, Substitution and uncertainty are plotted above.

4 Dataset

The data used for training the SVM model is borrowed from existing literature on fold-switching proteins and is provided in the tables below. In table S1, the columns PDB1 and PDB2 represent the PDB ID along with the chain ID for the two distinct secondary structures that were solved for the fold-switching protein.

4.1 Fold-switching Proteins Dataset

PDB1	PDB2	PDB1	PDB2	PDB1	PDB2
1ceeB	2k42A	3ejhA	3m7pA	4qdsA	2qqjA
1g2cF	5tpnA	3ewsB	3g0hA	4qhfA	4qhhA
1h38D	1qlnA	3gmhL	2vfxL	4rmbA	4rmbB
1k0nA	1rk4B	3hdeA	3hdfA	4rr2D	3l9qB
1mnmC	1mnmD	3ifaA	5et5A	4rwnA	4rwqB
1nqdA	1nqjB	3j7wB	3j7vG	4twaA	4ydqB
1ovaA	1jtiB	3j97M	1xtgB	4uv2D	4q79F
1qomB	1nocA	3j9cA	4h2aA	4wsgC	1svfC
1qs8B	1miqB	3jv6A	1zk9A	4y0mJ	4xwsD
1repC	2z9oB	4pyiA	4pyjA	4zrbC	4zrbH
1rkpA	2h44A	3kuyA	5c3iF	4zt0C	4cmqB
1uxmK	2namA	3m1bF	3lowA	5aoeB	5ly6B
1wyyB	5wrgC	3njqA	2pbkB	5b3zA	5bmyA
1x0gA	1x0gD	3o44A	1xezA	5c1vA	5c1vB
1xjtA	1xjuB	3qy2A	1qb3A	5ec5P	3zxgB
1xntA	3lqcA	3t1pA	1kctA	5ejbC	1wp8C
2a73B	3l5nB	3tp2A	5lj3M	5f3kA	5f5rB
2axzA	2grmB	3uyiA	3v0tA	5fhcJ	1eboE
2c1uC	2c1vB	3zwgN	4tsyD	5fluE	2uy7D
2ce7C	3kdsG	4a5wB	3t5oA	5hmgA	3ztjE
2gedB	1nrjB	4aanA	4aalA	5i2mA	5i2sA
2hdmA	2n54B	4ae0A	4ow6B	5ineA	3mkoA
2k0qA	2lelA	4b3oB	3meeA	5jzhA	5jztG
2lejA	2lv1A	4dxtA	4dxrA	5k5gA	2kb8A
2lqwA	2bzyB	4fu4C	4g0dZ	5keqF	1dzlA
2n0aD	2kkwA	4gqcC	4gqcB	5l35D	5l35G
2naoF	1iytA	4hddA	2lepA	7ahlE	4yhdG
2nntA	2mwfA	4j3oF	2jmrA	2KXOA	3RJ9C
2nxqB	1jfkA	4jphB	5hk5H	6Z4UA	7KDTB
2ougC	2lclA	4nc9C	4n9wA	4KSO	
2p3vA	2p3vD	4o0pA	4o01D	1F16	
2qkeE	5jytA	4phqA	2wcdX	8E6Y	

Table S1: 189 Metamorphic proteins that are used for training the model. PDB1 and PDB2 refer to the two different solved structures of the protein

4.2 Monomorphic Proteins Dataset

PDB ID	PDB ID	PDB ID	PDB ID
1O7N.B	5DPJ_1	1VF1.A	1PHN.A
1YCK.A	5DPG_1	2CHH.A	1W6N_1
2HBT.A	2ZMU_1	1FUS_1	2UYZ.A
1ELT.A	1RYP.L	3D80.A	1W0T_1
3RP2.A	1AUN.A	2INC.C	2M34_1
2BV4.A	3MGQ_3	2VML.A	3E2C.A
1NN6.A	2DYR_9	2DK7_1	3E86.A
1IAU.A	3MGQ_2	1KT6.A	2BC3.A
1SLT_1	2QWX.A	1BIO.A	2M7B_1
2W69.A	2JXW_1	1J7D.B	1UZV.A
1RVE.A	4UBP_1	2JE7.A	1KM4.A
2OQA.A	1AB9.B	2DWV_1	2HI3_1
2DYR_7	1A53.A	1ZBF.A	2GDG.A
1FON.A	2AWK_1	1IIU.A	3NAU_1
2HFT.A	2ELJ_1	1O6W_1	2HBA_1
1PMY.A	2JET.B	3DHI.C	4Z4M_1
3MGQ_1	8DFR.A	1AHC.A	4Z4K_1
2J96.A	2YS9_1	1HJ8.A	1GFL.A
1LM4_1	5DTX_1	3MGQ_4	2YSH_1
3EF4.A	1RYP.H	2JKH.A	7FD1.A
2A06.F	3SEB.A	3CHB.D	2GJD.A
2BUR.B	2VZX_1	1JJT.A	3B44.A
1TK7_1	3EIK.A	2VHK.A	2JV4_1
1QZP_1	1Z3Q.A	3D6M.B	1JOT.A
1EJX.B	3D6M.A	2O7M.A	5KEE_1
1KEQ.A	1CPC.A	1JBO.A	4EQP_1
1X8Q.A	1KMV.A	2UX7.A	5JOB_1
1YPH.C	1T32.A	1K55_1	2VI6_1
1K3Y.A	1N5N.A	1UBQ.A	2OV0.A
3ERX.A	2DYR.F	1PAZ.A	2RK3.A
2YSE_1	1WMV_1	2IH3_3	1UGX.A
1LQY.A	1WXC.B	3PCC.B	2VLQ.A
1MIJ_1	2CXQ_1	1CEX.A	2KPZ_1
1MQO.A	1UIZ.A	1BW5_1	1FME_1
2JOF_1	2YSB_1	3L4H_1	3ELN.A
1QMJ.A	4REX_1	1OPS.A	2Z8A.A
1Y71_1	2HPW_1	2JX8_1	2YSD_1
2ZO6_1	1T56.A	1WI3_1	3A03_1
1FIP.A	2KMU_1	1GB1_1	2NN8.A
1XME.B	1MVQ.A	1I5H_2	2YSG_1
2OGQ.A	2BV8.A	2A3D_1	1E85_1
3BWH.A	1F99.A	1KPF_1	2YSF_1
1XEO.A	2PGZ.A	3DR9.A	1I5H_1

2GH9_1	1LMB_3	2FKZ.A	3DJH.A
2DSC.A	5EJU_1	1GH0.A	253L.A
2FDJ.A	2BMO.B	1Y66_1	2DMV_1
5DPI_1	3DUI.A	5DTZ_1	3A02_1
5DPH_1	4HVF_1	5DU0_1	2DKO.B
5EHU_1	2UU8.A	2IB5_1	
5DY6_1	1MRJ.A	1L2H.A	

Table S2: 198 Monomorphic proteins that are used for training the model. The character after the dot/underscore refers to the chain ID of the protein

5 Optimizations

a. Rolling window width: The Rolling average window width is optimized by considering a range of values from 5 to 25 and picking the one that gives the best Mattew's Correlation coefficient (MCC) when applied to the training dataset. A six-fold cross-validation scheme is applied when the MCC value is calculated with each of the window widths. The validation accuracy and MCC score are calculated, and the window width that gives the best MCC score is selected. Here the columns are window width, Accuracy when a quadratic kernel is used, MCC when a quadratic kernel is used, Accuracy when a linear kernel is used, MCC when a linear kernel is used for the SVM.

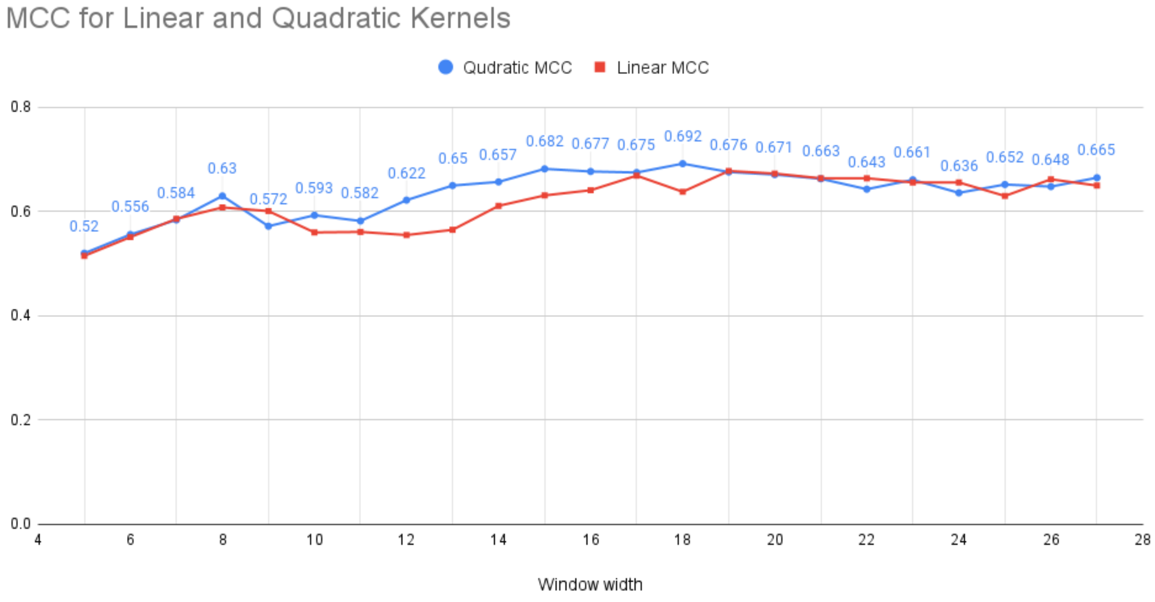

Figure S4.j: The MCC values for each window width is plotted and the top two window widths (8,18) are selected later to be used for calculating the diversity metrics

b. Substitution Matrix: Similarly to optimizing the window width, the substitution

Window width	Quad Acc	Quad SVM MCC	Lin SVM Acc	Lin SVM MCC
5	77.2	0.52	75.1	0.515
6	77.8	0.556	77.5	0.551
7	79.1	0.584	79.3	0.586
8	81.4	0.63	81.5	0.608
9	78.5	0.572	80.1	0.601
10	79.6	0.593	78	0.56
11	78.8	0.582	78	0.561
12	80.8	0.622	77.7	0.555
13	82.1	0.65	78.2	0.565
14	82.6	0.657	80.57	0.611
15	83.9	0.682	81.6	0.631
16	83.6	0.677	82.1	0.641
17	83.6	0.675	83.4	0.669
18	84.4	0.692	81.8	0.638
19	83.6	0.676	83.9	0.678
20	83.4	0.671	83.6	0.673
21	82.9	0.663	83.1	0.664
22	81.8	0.643	83.1	0.664
23	82.8	0.661	82.8	0.656
24	81.5	0.636	82.8	0.656
25	82.3	0.652	81.5	0.63
26	82.3	0.648	83.1	0.662
27	83.1	0.665	82.6	0.65

Table S3: The Accuracy and MCC measures are provided for each of the value of averaging window width ranging from 5 to 27. These measures are calculated by performing a 6-fold cross-validation on the training dataset.

matrix is optimized using a range of parameters the form shown below:

$$\begin{bmatrix} \text{ref/replace} & \text{H/I/G} & \text{E/B} & \text{L/C/S} & \text{T} \\ \text{H/I/G} & 0 & a & b & c \\ \text{E/B} & a & 0 & b & d \\ \text{L/C/S} & b & b & 0 & 0 \\ \text{T} & c & d & 0 & 0 \end{bmatrix}$$

The values for a,b,c and d were sampled first in a coarse manner, where the values of all the 4 parameters range from 0 to 1 with a step of 0.2 for each one. Then once a substitution matrix that gave the maximum MCC value for the substitution score is found, the sampling is made much finer with a step value of 0.05 for each of the parameter. That way we arrived at a final substitution matrix as shown (d=0):

$$\frac{1}{7}\begin{bmatrix} \text{ref/replace} & \text{H/I/G} & \text{E/B} & \text{T} \\ \text{H/I/G} & 0 & 0.7 & 0.3 \\ \text{E/B} & 0.7 & 0 & 1 \\ \text{T} & 0.3 & 1 & 0 \end{bmatrix}$$

c. Fragment Size: The fragment size is optimized via trial and error. For the training dataset. Fragment picking was performed on the training dataset with the fragment

sizes: 6, 7 and 8. When fragment picking is performed with a fragment size of 8, the number of hits, i.e. proteins containing the identical fragment, was quite low with the database currently in use. While this does not give us enough data to work with, using fragment size 6 incurred the problem of the fragment secondary structure diversity, being all over the place. The 6-mers did not seem to truly represent the diversity taken by the protein in question. Therefore the fragment size is set to 7 and the high accuracy in prediction seems to validate the choice of picking 7-mers.

d. Middle Residue: After fragment picking, the residue that was taken into consideration for the secondary structure analysis is the middle residue (4th residue of the 7-mer fragments). But upon considering other residues, there was a small change in accuracy measures. Although the accuracy measures when whichever residue is taken into consideration did not vary beyond a significant number standard deviation, we still chose to work with the one that gave the highest MCC score. Table S4 shows the variation of accuracy when the fragment in consideration is changed from the 2nd residue to the 6th residue of the 7-mer. Accordingly, we chose the final model to work with the 3rd residue.

Measure	2nd RSD	3rd RSD	4th RSD	5th RSD	6th RSD
TPR	0.82 (0.08)	0.79 (0.08)	0.78 (0.05)	0.87 (0.04)	0.79 (0.02)
SPC	0.85 (0.06)	0.90 (0.06)	0.86 (0.07)	0.74 (0.12)	0.89 (0.05)
PRE	0.84 (0.05)	0.89 (0.08)	0.85 (0.07)	0.77 (0.08)	0.87 (0.05)
ACC	83.42 (4.3%)	84.75 (6.0%)	81.87 (4.4%)	80.55 (6.3%)	83.93 (2.7%)
F1	0.83 (0.04)	0.83 (0.08)	0.81 (0.04)	0.82 (0.05)	0.83 (0.02)
MCC	0.673 (0.08)	0.698 (0.12)	0.639 (0.09)	0.619 (0.12)	0.678 (0.05)

Table S4: The Accuracy measures for the residue in consideration starting from the 2nd residue to the 6th residue of the 7-mer fragment.

e. Support Machine Vector Model: An SVM model with a quadratic kernel is chosen for making predictions on data the training model has not encountered before. Support vector machine is chosen as they achieve good performance on many classification tasks. Kernels make SVMs more flexible and able to handle nonlinear problems, and here as we are using a quadratic kernel, we do not have to worry about the model complexity. As we have a relatively small dataset to train with, this restricts the choices of many machine learning models we could use. A quadratic SVM model gives us good accuracy while still maintaining a low model complexity.
The hyperparameters box constraint and Kernel scale were optimized using a Bayesian optimization technique in MATLAB.

References

[1] Lauren L. Porter and Loren L. Looger. Extant fold-switching proteins are widespread. *Proceedings of the National Academy of Sciences*, 115(23):5968–5973, June 2018. Publisher: Proceedings of the National Academy of Sciences.

[2] Nanhao Chen, Madhurima Das, Andy LiWang, and Lee-Ping Wang. Sequence-Based Prediction of Metamorphic Behavior in Proteins. *Biophysical Journal*, 119(7):1380–1390, October 2020.
